# Liquid-to-solid phase transition of *oskar* ribonucleoprotein granules is essential for their function in *Drosophila* embryonic development

**DOI:** 10.1016/j.cell.2022.02.022

**Published:** 2022-04-14

**Authors:** Mainak Bose, Marko Lampe, Julia Mahamid, Anne Ephrussi

**Affiliations:** 1 Developmental Biology Unit, European Molecular Biology Laboratory, Heidelberg 69117, Germany; 2 Advanced Light Microscopy Facility, European Molecular Biology Laboratory, Heidelberg 69117, Germany; 3 Structural and Computational Biology Unit, European Molecular Biology Laboratory, Heidelberg 69117, Germany

**Keywords:** ribonucleoprotein granules, RNP granules, biomolecular condensates, phase separation, material properties, *oskar* mRNA, embryonic development, RNA localization, translation control

## Abstract

Asymmetric localization of *oskar* ribonucleoprotein (RNP) granules to the oocyte posterior is crucial for abdominal patterning and germline formation in the *Drosophila* embryo. We show that *oskar* RNP granules in the oocyte are condensates with solid-like physical properties. Using purified *oskar* RNA and scaffold proteins Bruno and Hrp48, we confirm *in vitro* that *oskar* granules undergo a liquid-to-solid phase transition. Whereas the liquid phase allows RNA incorporation, the solid phase precludes incorporation of additional RNA while allowing RNA-dependent partitioning of client proteins. Genetic modification of scaffold granule proteins or tethering the intrinsically disordered region of human fused in sarcoma (FUS) to *oskar* mRNA allowed modulation of granule material properties *in vivo*. The resulting liquid-like properties impaired *oskar* localization and translation with severe consequences on embryonic development. Our study reflects how physiological phase transitions shape RNA-protein condensates to regulate the localization and expression of a maternal RNA that instructs germline formation.

## Highlights

• *oskar* RNP granules in the developing oocyte are solid-like condensates
• *oskar* RNP granules undergo liquid-to-solid phase transition *in vitro*
• The liquid phase incorporates mRNA, while the solid phase enriches specific proteins
• Perturbing the solid state impairs *oskar* localization, translation, and development

## Graphical abstract

**Figure:**
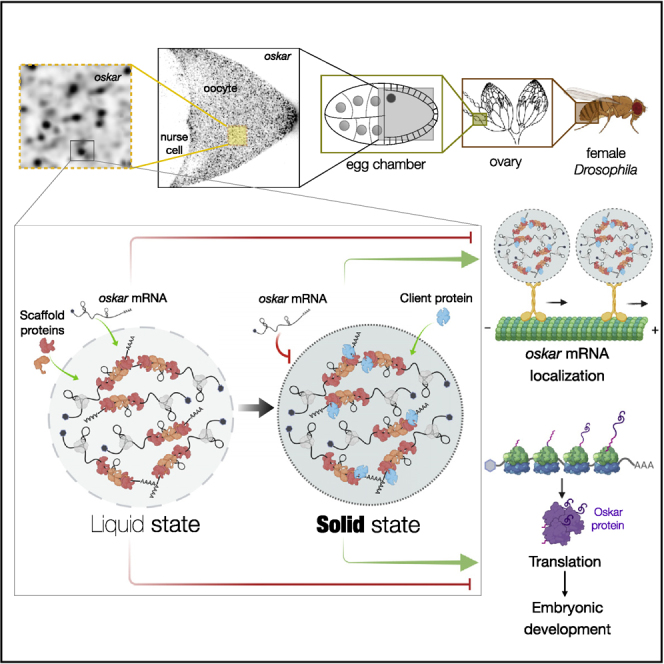

## Teaser

Liquid-to-solid phase transition of *oskar* RNP granules ensures *Drosophila* embryonic development by regulating *oskar* mRNA localization and translation.

## Introduction

Asymmetric localization of maternal RNAs in the developing oocyte is essential for embryonic axis formation and cell fate specification in many organisms (Becalska and Gavis, 2009; Besse and Ephrussi, 2008; Buxbaum et al., 2015; Martin and Ephrussi, 2009). In *Drosophila*, *oskar* mRNA encodes the posterior determinant, Oskar protein. Posterior accumulation of Oskar is achieved by active transport of *oskar* mRNA as diffraction-limited granules on a polarized microtubule network during mid-oogenesis, in a two-step transport process involving dynein and kinesin motors (Brendza et al., 2000; Clark et al., 2007; Jambor et al., 2014; Little et al., 2015; Zimyanin et al., 2008). Importantly, *oskar* mRNA is translationally repressed prior to localization, preventing ectopic production of Oskar protein (Ephrussi et al., 1991; Ephrussi and Lehmann, 1992; Kim-Ha et al., 1995). The translated Oskar protein nucleates pole plasm assembly, which induces abdominal patterning and germline formation in the embryo.

Inside cells, mRNAs interact with proteins to form RNP complexes, in which protein composition is dynamically remodeled during the mRNA life cycle (Moore, 2005). At high local concentrations, individual RNPs can condense into higher-order assemblies by virtue of multivalent protein-protein, RNA-protein, and/or RNA-RNA interactions. These mesoscale assemblies, referred to as RNP granules, belong to the expanding class of membraneless compartments or biomolecular condensates (Tauber et al., 2020; Van Treeck and Parker, 2018), some of which form by liquid-liquid phase separation (LLPS) (Banani et al., 2017; Shin and Brangwynne, 2017). The collective behavior of RNPs in the condensed state confers emergent properties to the granules that the individual RNPs lack, and that may also evolve with time (Alberti, 2017; Alberti et al., 2019). For example, reconstitution experiments with the stress granule protein fused in sarcoma (FUS) have shown that FUS droplets assemble by LLPS into spherical condensates that can mature with time into a solid non-dynamic state, a phenomenon described as aging (Patel et al., 2015). Condensate aging is physiologically pertinent as evident from the *C. elegans* pericentriolar matrix (PCM), which exhibits distinct liquid-like and solid-like physical states at different stages of the embryonic cell cycle (Mittasch et al., 2020; Woodruff et al., 2017, 2018). Thus, cells harness different condensate properties to achieve specific functions.

We report that *oskar* granules are RNA-protein condensates that behave as solids *in vivo* and *in vitro*, unlike the vast majority of liquid-like RNP granules described to date (Banani et al., 2017; Brangwynne et al., 2009, 2011; Fujioka et al., 2020; Shin and Brangwynne, 2017; Wippich et al., 2013). *In vitro* reconstitution of *oskar* 3′UTR with scaffold granule proteins leads to the formation of amorphous, spherical, and dynamic condensates that rapidly mature into a solid state. This liquid-to-solid transition is physiologically essential, as perturbing the solid state *in vivo* impaired RNA localization and translation, linking regulation of granule material properties to RNA post-transcriptional control.

## Results

### *oskar* RNP granules in the oocyte are spherical solid-like assemblies

**Figure 1:**
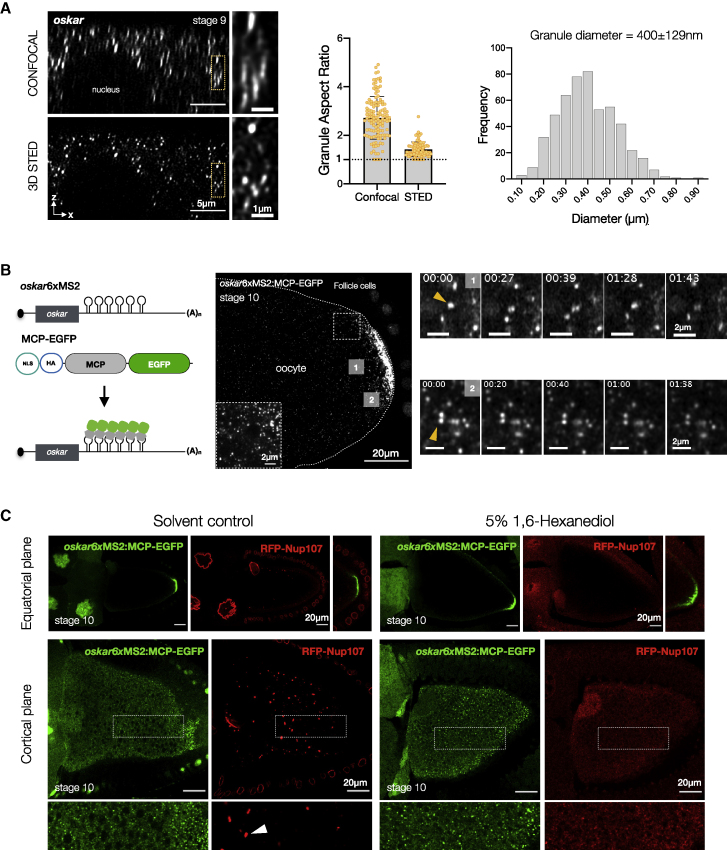
*oskar* RNP granules are spherical, solid-like assemblies (A) Confocal and STED stacks in XZ plane, with *oskar* (grayscale) detected by single-molecule fluorescence *in situ* hybridization (smFISH) in wild-type (*w*^*1118*^) egg chambers. Center: comparison of granule aspect ratio. Error bars represent SD. Right: distribution of granule diameter as measured by STED. (B) Schematic of *oskar* mRNA *in vivo* labeling. A representative stage 10 egg chambers with *oskar* granules in grayscale. Live imaging of granules (min:sec) was performed on a cortical region approximately 15–20 μm from the posterior pole (1) and on granules at the posterior pole (2). A total of ∼70 pairs and clusters of granules were observed from time-lapse movies of six oocytes. (C) Representative images of egg chambers expressing RFP-Nup107 and *oskar*6xMS2-MCP-EGFP after treatment with solvent control or 5% 1,6-hexanediol for 15 min. White arrowheads, annulate lamellae. See also Figure S1; Video S1.

Proteins involved in transport and/or translational control associate with *oskar* mRNA. The bona fide RNP components Bruno and PTB (polypyrimidine tract binding protein) have been shown to oligomerize on the *oskar* 3′UTR, forming higher-order complexes (Besse et al., 2009; Chekulaeva et al., 2006). *oskar* mRNA can dimerize by virtue of a stem-loop structure in the 3′UTR (Jambor et al., 2011). These findings suggest that multivalent interactions between *oskar* and associated proteins promote the formation of higher-order transport granules and prompted us to investigate the potential role of biomolecular condensation in granule assembly and function. A liquid condensate assumes a spherical shape due to surface tension (Widom, 1988). This shape criterion determined by observations made with light microscopy has been central in assessing whether granules assemble via LLPS (Alberti et al., 2019; Hyman et al., 2014). However, *oskar* granules in the oocyte are diffraction-limited point sources, which precluded characterization of their shape by conventional confocal microscopy. We therefore resorted to 3D STED (stimulated emission depletion) super-resolution imaging with near isotropic resolution. This unequivocally shows that *oskar* granules are spherical, with an aspect ratio ∼1 and a Gaussian distribution of sizes (Figure 1A and STAR Methods), consistent with the notion that assembly *in vivo* is driven by LLPS.

Upon contact, liquid-like condensates typically fuse and rearrange into a spherical structure (Brangwynne et al., 2009; Hyman et al., 2014). To investigate the dynamic behavior of *oskar* RNP granules, we tagged *oskar* with EGFP (enhanced green fluorescent protein) using the MCP-MS2 (MS2 bacteriophage coat protein) tethering system (Bertrand et al., 1998). Imaging in live egg chambers near the cortical surface visualized occasionally directed transport on microtubule tracks, in addition to diffusive movements due to Brownian motion and cytoplasmic flows. Interestingly, two touching granules did not fuse and relax into one within the timescale of imaging (4 min, Figure 1B (1); Video S1). At the posterior pole, granules that are presumably anchored also did not fuse despite their high local concentration, indicating that *oskar* granules are not liquid-like (Figures 1B (2) and S1A; Video S1).

**Figure S1:**
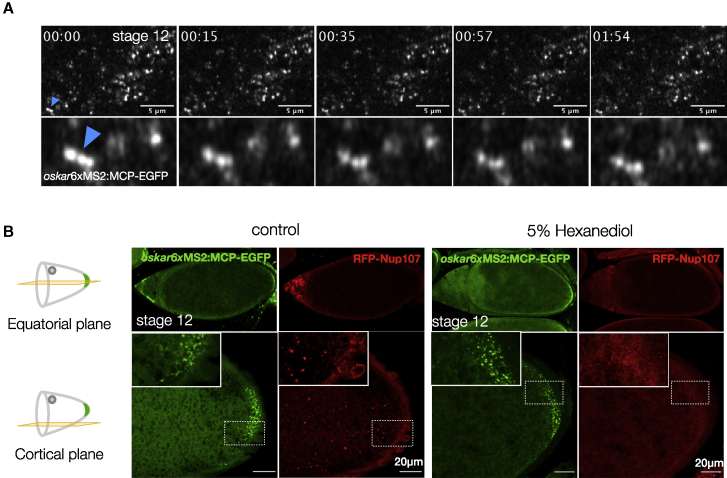
*oskar* granules maintain their solid-like behavior in late oogenesis, related to Figure 1 (A) Live imaging of MCP-EGFP tethered *oskar* granules performed on a cortical region at the posterior pole in a stage 12 egg chamber. The time point (min:sec) is indicated at the top of each frame. Arrowhead indicates a cluster of granules that is zoomed below. Also refer to Video S1. (B) Representative images of stage 12 egg chambers expressing RFP-Nup107 and *oskar*6xMS2-MCP-EGFP after treatment with solvent control or 5% 1,6-hexanediol for 15 min.

Video S1. Fluorescence time-lapse movie of *oskar* granules labelled with MCP-EGFP (grayscale) near the posterior pole and at the posterior cortex, Scale 2 μm, related to Figures 1 and S1

A liquid phase is susceptible to dissolution upon dilution (Putnam et al., 2019). Extrusion of ooplasm into a physiological buffer does not dissolve *oskar* granules, confirming their non-liquid properties (Gaspar and Ephrussi, 2017; Gáspár et al., 2017a). Furthermore, treatment of egg chambers with 1,6-hexanediol, which can perturb weak multivalent interactions in LLPS and is used as a probe to distinguish liquid from solid condensates (Kroschwald et al., 2017), dissolved phase-separated precursors of nuclear envelopes labeled with red fluorescent protein (RFP)-Nup-107 (Hampoelz et al., 2019) but had no effect on *oskar* granules (Figures 1C and S1B). Taken together, these data suggest that *oskar* RNP granules *in vivo* are phase-separated solid-like assemblies.

### Bona fide *oskar* granule proteins are RNA-binding proteins with structural disorder

Genetic studies identified several RNA-binding proteins (RBPs) that associate with *oskar* mRNA and engage in diverse processes, including RNP transport and translation repression (Besse et al., 2009; Huynh et al., 2004; Kim-Ha et al., 1995; Snee et al., 2008; Yano et al., 2004). The proteins Bruno, Hrp48, and PTB bind specific sequences along the RNA and regulate its translation. Moreover, Bruno and PTB form higher-order structures with *oskar* 3′UTR (Besse et al., 2009; Chekulaeva et al., 2006). We therefore hypothesized that these bona fide granule proteins may form the scaffold of the granule and contribute to condensation. However, as the germline is a syncytium, bulk biochemistry does not reveal where (nurse cells or oocyte) and at what stoichiometries these proteins associate with *oskar*.

**Figure S2:**
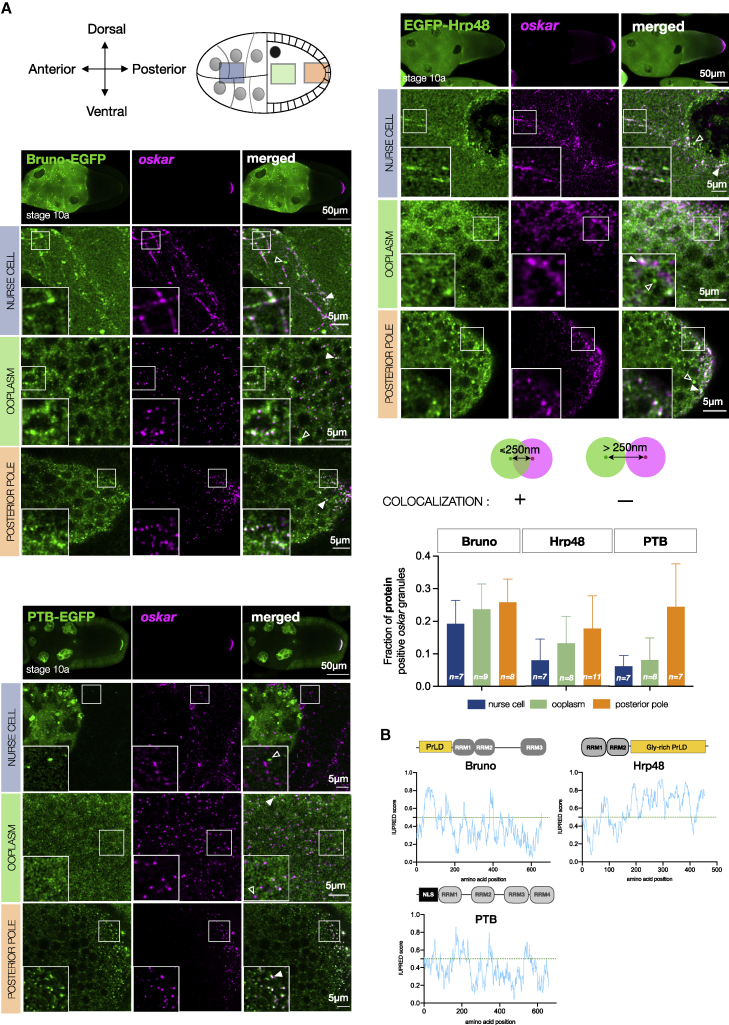
Association of *oskar* RNA with bona fide granule proteins in the egg chamber, related to Figure 2 (A) *oskar* mRNA association with three bona fide granule proteins. smFISH for *oskar* mRNA (magenta) and the respective proteins (green). A maximum intensity projection of a z stack of 1 μm is shown for nurse cell, ooplasm, and posterior pole (cortical plane). For Bruno, BrunoFL-EGFP was conditionally expressed in the germline in an endogenous Bruno-deficient genetic background (*aret*PA62/*aret*^*CRISPR*null^). For Hrp48, EGFP-Hrp48 FL was expressed in the germline in an *hrp48*-RNAi background (refer to Figure S6 for knockdown efficiency). For PTB, a homozygous EGFP-trap line was used in which both alleles of PTB bear the GFP insertion. Insets are marked with a white boxes. White arrowheads, colocalized spots; empty arrowheads, absence of colocalization. Quantification of colocalization frequency in the three cellular compartments using an object-based approach (see STAR Methods). smFISH probe set used for *oskar* RNA (5′ to 3′): gatccatcagcgtaaatcg, ccaacttaatactccagactcg, ccagaacagatagggttcc, tcgttgattagacaggagtg, acaatagttgcccagcgg, tttgttagaatcggcaccaa, gcatattgtgcatctccttga, ctcgatctgaaccaaaggc, ataatgtccaccgatccga, gacgatgatctgagtaccc, agtccggatacacaaagtcc, cattcgggcgagatatagca, catcgcccataagcggaaag, agataggcatcgtaatccgag, tcgtcagcagagaatcgttg, gtcatttcgtggcgtctct, gctttgggttctgcagct, gagccaaattgattggttcctc, gctgtagatgttgatggg, gcatttacgctggcttgc, aattatcctggtagcaccag, gtttgaagggattcttccag, aggtgctcgtggtatgttc, tagtcgctggtgcgctct, agcaccatatccaggagg, cgttcttcaggctcgctt, aagatccgcttaccggac, ctgcactcagcggtcaca, ggaatggtcagcaggaaa, cgtcacgttgtcgtgcag, aaatggattgcccgtcag, cttgatgctcgatatcgtga, tgggcgtggctcagcaata, cgcgcacctcactatcta, atattcctcgcgcacgga, atagttgctctcgatgatgg, tgttctcgctggtgttgc, gttgtaggtgatttccttgg, tctgagtggacgagaagag, gctacgacttgcaactgc, gagttcatgggccaccaa, cttccacaactccggcaa. (B) Domain architecture of the three *oskar* granule proteins with disorder prediction using IUPred (Mészáros et al., 2018).

**Figure 2:**
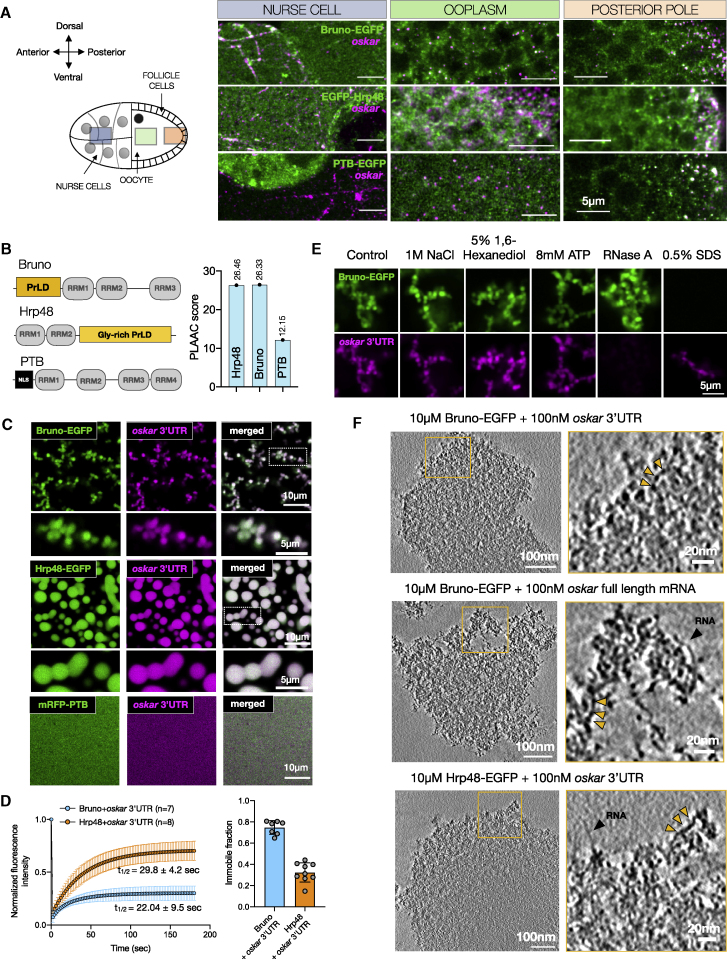
*In vitro* reconstituted minimal *oskar* RNP condensates recapitulate properties of *in vivo* RNP granules (A) *oskar* mRNA (magenta) association with three bona fide granule RBPs (green) in the nurse cell cytoplasm, ooplasm, and posterior pole of stage 10 egg chambers. (B) Domain architecture and PLAAC score of the three RBPs. RRM, RNA recognition motif; NLS, nuclear localization signal. (C) Condensates formed with 100 nM *oskar* 3′UTR-atto633 (magenta) and 10 μM of the indicated RBPs (green) imaged with confocal microscopy. (D) Quantification of fluorescent recovery after photobleaching (FRAP) kinetics and immobile fractions of condensates assembled with 100 nM *oskar* 3′UTR and 10 μM Bruno-EGFP or Hrp48-EGFP. Error bars, SD; N, number of movies. (E) Bruno-EGFP (green)-*oskar* 3′UTR (magenta) condensates subjected to the indicated treatments after 30 min of aging. Images acquired under identical microscope settings. (F) 4 nm-thick tomographic slices of condensates formed under the indicated conditions and plunge frozen after 30 min of aging. Yellow arrowheads, “beads on a string” structures; black arrowheads, putative naked RNA molecules. See also Figures S2 and S3; Video S2.

We therefore used imaging to determine where the candidate proteins associate with *oskar* (Figures 2A and S2A). Bruno colocalized with *oskar* in the nurse cell cytoplasm on track-like structures, presumably corresponding to the microtubule network (Zimyanin et al., 2008; Gáspár et al., 2017a). The association was maintained in the ooplasm and posterior pole. Hrp48 was diffuse in the nurse cell cytoplasm, with occasional enrichment with *oskar* on track-like structures. Granular appearance and *oskar* association became prominent in the ooplasm and posterior pole. PTB, in contrast, was largely nuclear in nurse cells with no obvious colocalization with *oskar* but associated with *oskar* markedly at the posterior pole, indicating sequential recruitment of proteins to *oskar* granules.

RBPs with prion-like domains (PrLDs) play key roles in granule formation by promoting multivalent interactions involving RNA, folded protein domains, PrLDs of their own, and/or of other proteins (Protter et al., 2018; Li et al., 2012). We therefore asked whether *oskar* RBPs possess unstructured domains. Using the prion prediction algorithm PLAAC (Lancaster et al., 2014), we noted that Bruno has an N-terminal PrLD with an over-representation of Ser and Asn. Scoring similar to Bruno, Hrp48 possesses a 200-residue-long C-terminal PrLD enriched in Ser and Gly. PTB, which comprises four RRMs (RNA recognition motifs), lacks substantial disorder (Figures 2B and S2B). Thus, the *oskar* granule proteins Bruno and Hrp48 may be sufficiently disordered to drive LLPS. PTB, on the other hand, could contribute to condensation through multivalency via its multiple RRMs. We therefore tested the three proteins for their propensity to promote *oskar* condensate formation.

### *In vitro* reconstituted minimal *oskar* RNP condensates undergo liquid-to-solid phase transition

**Figure S3:**
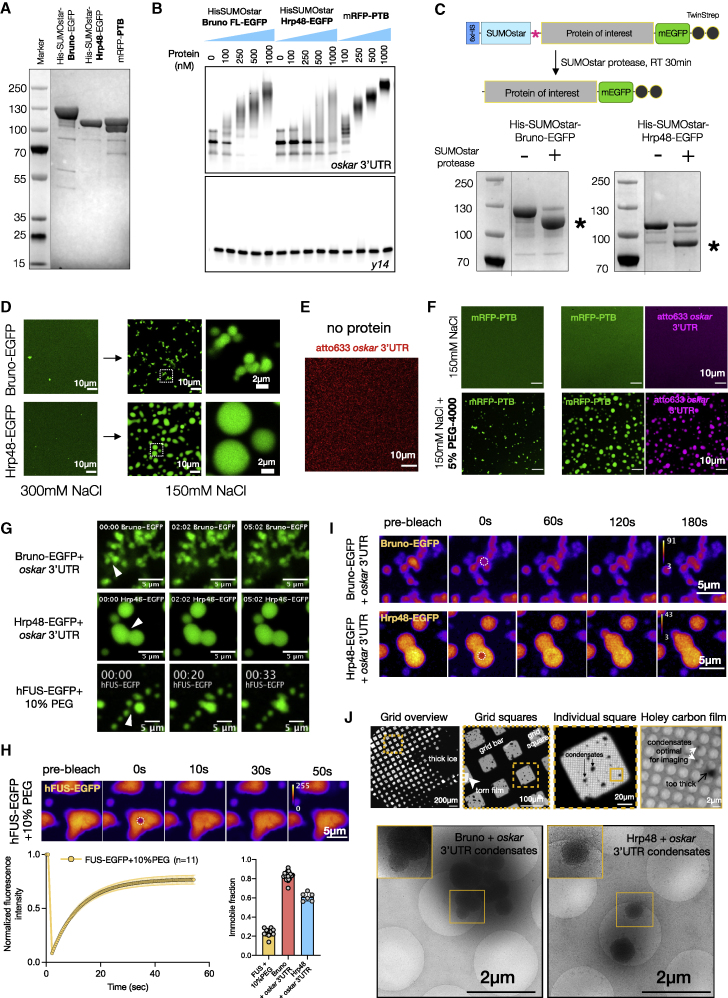
*In vitro* condensate assembly with *oskar* 3′UTR and granule RBPs, related to Figure 2 (A) Coomassie-stained SDS-PAGE gel of the purified three granule proteins from insect cells. (B) EMSA demonstrating the intrinsic affinities of the purified granule proteins for *oskar* 3′UTR RNA. 50 nM atto633-labeled *oskar* 3′UTR or *y14* (control RNA) was used with the indicated concentrations of the proteins, and the reaction was resolved on an agarose gel. (C and D) Schematic representation of the *in vitro* condensate assembly assay for Bruno and Hrp48. 10 μM of tagged protein incubated with 1 U of SumoStar protease for 30 min at room temperature followed by SDS-PAGE or imaging. SDS-PAGE shows the efficiency of tag cleavage in assay buffer with 300 mM NaCl; ∗ indicates the cleaved protein band (C). Tag cleavage does not induce condensate formation in 300 mM NaCl buffer. Exchange to 150 mM NaCl buffer triggers LLPS of Bruno and Hrp48 (D). (E) 100 nM *oskar* 3′UTR-atto633 (red) does not self-assemble into condensates in absence of protein under the same conditions. Note that the laser power used for imaging was five times higher than for other conditions. (F) Molecular crowding promotes condensate formation of *oskar* 3′UTR with PTB. 10 μM RFP-PTB (green) is soluble in 150 mM NaCl buffer in absence or presence of *oskar* 3′UTR (magenta). Addition of 5% (w/v) PEG-4000 induces formation of spherical condensates of PTB alone and with *oskar* 3′UTR. (G) Condensates formed with 10 μM Bruno-EGFP or Hrp48-EGFP (green) and 100 nM *oskar* 3′UTR do not fuse and relax like liquid droplets, unlike hFUS-EGFP condensates assembled with 8-μM hFUS-EGFP (without RNA) in presence of 10% PEG-4000 (Patel et al., 2015). (H) FRAP of hFUS-EGFP condensates assembled with 8 μM hFUS-EGFP (without RNA) and 10% PEG-4000 (Patel et al., 2015). The bleached region of interest (ROI) is marked with a dotted circle. Bottom right: quantification of immobile fractions of hFUS droplets, and Bruno-EGFP (10 μM) or Hrp48-EGFP (10 μM) condensates assembled with 100 nM *oskar* 3′UTR in FRAP assays where fluorescence recovery was recorded up to 1 min after bleaching. (I) FRAP movie snapshots of 10 μM of Bruno or Hrp48 (heatmap) condensates assembled with 100 nM *oskar* 3′UTR (unlabeled) at indicated time points. The bleached ROI is marked with a dotted circle. Quantification is provided in Figure 2D. (J) Top panel: cryo-EM image of Bruno-*oskar* 3′UTR condensates deposited on a holey carbon EM grid. Leftmost panel shows a grid map with varying ice thickness, and yellow dotted box represents grid squares enlarged on the right. Within an individual square, condensates are indicated by black arrows. Small condensates deposited in holes amenable to tilt series acquisition are marked with white arrows, whereas black arrow marks a condensate on the support film that is too thick to be imaged. Bottom panel: cryo-EM overview images of Bruno-*oskar* 3′UTR (left) and Hrp48-*oskar* 3′UTR (right) condensates on the EM grid holey-support film. Left panel shows a cluster of spherical condensates too thick for acquiring tilt series; inset shows an enlarged view of spherical condensates. Right panel shows two spherical condensates; inset shows an enlarged view of one that is suitable for tilt series acquisition.

We purified EGFP-tagged full-length Bruno and Hrp48 using a solubility tag (6 × His-SumoStar) and monomeric RFP (mRFP)-PTB with a 6 × His tag from insect cells (Figure S3A). Electrophoretic mobility shift assay (EMSA) confirmed that all three proteins directly and specifically bind *oskar* 3′UTR *in vitro*, forming higher-order oligomers (Figure S3B). Cleavage of the solubility tag from Bruno and Hrp48, coupled with buffer exchange to physiological salt concentration (150-mM NaCl), triggered self-assembly of both proteins into spherical condensates (Figures S3C and S3D). Moreover, the proteins co-condensed with *in vitro* transcribed *oskar* 3′UTR (Figure 2C). Bruno-*oskar* 3′UTR condensates were ∼1 μm in diameter and tended to stick to each other, while Hrp48-*oskar* 3′UTR formed larger droplets. Notably, under the same conditions, *oskar* 3′UTR alone did not self-assemble (Figure S3E). mRFP-PTB was soluble (Figure 2C), and only formed condensates with the addition of a crowding agent, in the presence or absence of *oskar* 3′UTR (Figure S3F).

We did not observe fusion when *oskar* 3′UTR condensates with Bruno or Hrp48 settled on the glass surface (Figure S3G). In contrast, condensates formed by hFUS-EGFP in the same experimental setup fused as reported previously (Figure S3G) (Patel et al., 2015). Unlike FUS, recovery from photobleaching was negligible in Bruno-*oskar* 3′UTR condensates. Hrp48 condensates showed intermediate recovery kinetics (Figures 2D, S3H, and S3I). The spherical shape, but the lack of fusion and fluorescent recovery after photobleaching (FRAP), indicates a rapid liquid-to-solid phase transition *in vitro*, with Bruno showing a larger immobile fraction compared with Hrp48. Owing to the diffraction-limited size and the high local abundance at the posterior pole, FRAP on *oskar* granules *in vivo* could not be performed. *In vitro* Bruno-*oskar* 3′UTR condensates were stable under conditions that dissolve liquid droplets (Figure 2E) (Putnam et al., 2019). 0.5% SDS dissolved Bruno from the condensates, confirming that Bruno does not transition into amyloids but forms a stable solid-like phase. Signal from residual RNA (Figure 2E) indicates the formation of RNA-RNA interactions induced upon Bruno-driven condensation, as *oskar* 3′UTR alone did not self-assemble under identical conditions. Cryo-electron tomography (cryo-ET) confirmed that *in vitro* condensates, which are below the diffraction limit of a conventional light microscope, are spherical (Figure S3J). Cryo-ET of the protein condensates formed in the presence of either the 3′UTR or full-length *oskar* mRNA revealed that the solid-like condensates are amorphous. This indicates that the solid phase does not arise from large-scale molecular rearrangement into fibrils but likely occurs by jamming of component molecules in a glass state (Figure 2F; Video S2) (Jawerth et al., 2020).

Video S2. Tomographic reconstruction of condensate formed with 10 μM Bruno-EGFP and 100 nM *oskar* 3'UTR (left) or 100 nM full length *oskar* mRNA (center), 10 μM Hrp48-EGFP and 100 nM *oskar* 3'UTR (right), related to Figure 2 Scale bar 100 nm, each slice is 4 nm thick.

The material properties of the minimal *in vitro* condensates are reminiscent of the solid-like nature of *oskar* granules in the oocyte and suggest that a liquid-to-solid phase transition follows *oskar* RNP assembly *in vivo*. We could not detect a liquid-like state of the granules *in vivo*. It is possible that rapid hardening to a solid state *in vivo* arrests the granules as submicron particles precluding fusion into micron-scale assemblies.

### The liquid phase is essential for incorporation of *oskar* mRNA *in vitro*

**Figure 3:**
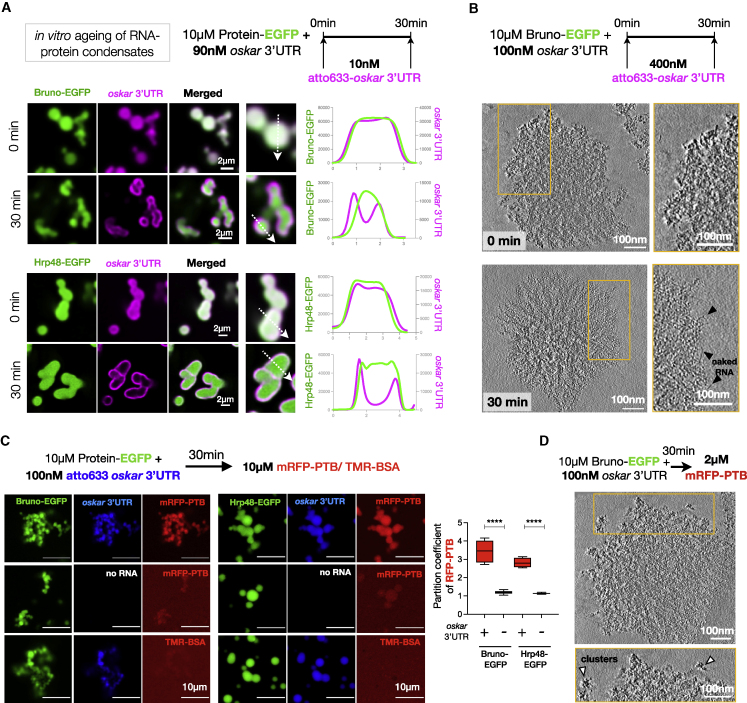
*In vitro* reconstituted *oskar* RNP condensates are selectively permeable (A) Scheme of the *in vitro* condensate ageing assay. Single confocal slices and representative line profiles (dotted arrows) shown. (B) 4 nm-thick tomographic slices of condensates at 0 and 30 min after addition of 400 nM atto633-*oskar* 3′UTR. Black arrowheads, naked RNA strands. (C) Condensates assembled with *oskar* 3′UTR-atto633 (blue) and EGFP-tagged Bruno or Hrp48 (green). mRFP-PTB (red) or TMR (tetramethyl rhodamine)-BSA (red) was added after 30 min. Single confocal slices shown; partition coefficient of mRFP-PTB calculated from 10 fields of view. (D) Tomographic slices (4 nm thick) of 2 μM mRFP-PTB added to 30 min aged Bruno-*oskar* 3′UTR condensates. White arrowheads, protein clusters. See also Figure S4; Video S3.

**Figure S4:**
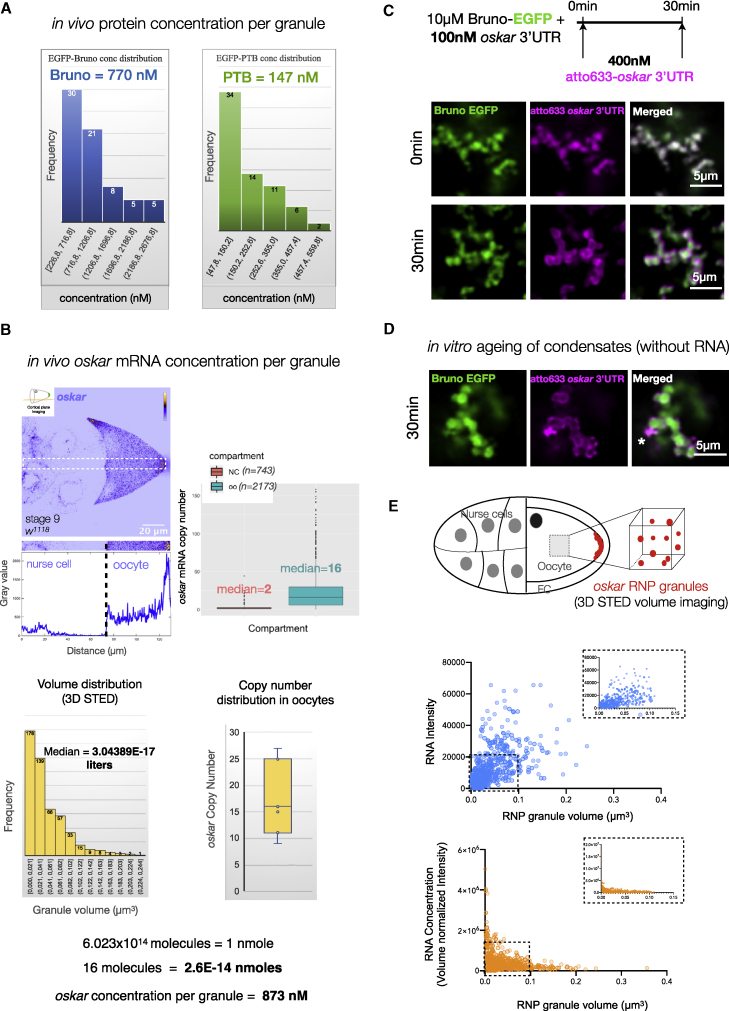
The liquid phase is essential for incorporation of *oskar* mRNA *in vitro*, related to Figure 3 (A) Quantification of *in vivo* protein concentrations per granule. GFP-trap lines of Bruno and PTB were used and absolute concentrations calculated based on a calibration curve of recombinant EGFP imaged under identical conditions in the same imaging session (Xing et al., 2020). Numbers in the histogram refer to the mean number of granules grouped under the indicated range of concentration. (B) For *in vivo oskar* RNA concentration per granule, *w*^*1118*^ egg chambers were stained for *oskar* by smFISH, and *oskar* copy number per granule in the oocyte compartment was calculated. A representative *oskar* smFISH image of a cortical plane acquisition done in “photon-counting mode” to avoid saturation of the signal in the oocyte. The intensity profile of the boxed area indeed shows the increase in *oskar* signal intensity along the AP axis. Granule volume obtained from 3D STED experiments was plotted, and absolute molar concentration of *oskar* RNA per granule was then derived based on average granule volume. Numbers in the histogram refer to the number of granules grouped under the indicated range of volume. (C) Representative light microscopy single plane confocal images of experimental conditions used for cryoelectron tomography in Figure 3B. Images were acquired and processed independently. (D) Condensates with Bruno alone preclude incorporation of *oskar* 3′UTR. Condensates were assembled with Bruno alone in 150 mM NaCl assay buffer. 10 nM atto633 labeled *oskar* 3′UTR RNA was added at 30 min of condensate aging. Note that new condensates formed after RNA addition show colocalization of the RNA and protein (marked by ^∗^). (E) Plot of mRNA intensity versus granule volume of *oskar* RNP granules measured by 3D STED on *w*^*1118*^ egg chambers probed for *oskar* mRNA by smFISH. Intensity of *oskar* mRNA signal (top plot) was normalized by granule volume to derive RNA concentration per granule, which does not increase with increase in granule volume.

Condensation might allow the packaging of several *oskar* RNPs within a granule for efficient posterior localization. Therefore, we hypothesized that the transient liquid state is required for RNA incorporation. To test this, we assembled Bruno-*oskar* 3′UTR (100:1 molar ratio) condensates *in vitro* and then added fluorescently labeled *oskar* 3′UTR. When labeled RNA was added at 0 min, its fluorescent signal overlapped with Bruno condensates. Fluorescent RNA added after 30 min instead formed a shell on the condensate surface (Figure 3A). Lowering the ratio of protein to RNA concentrations to 20:1, to recapitulate stoichiometries closer to those we measured in oocytes, resulted in a similar exclusion of labeled RNA at 30 min (Figures S4A–S4C). Aged Bruno condensates formed in the absence of RNA also excluded RNA added at 30 min, confirming that the exclusion is a consequence of the physical properties of Bruno in an aged, condensed state and not due to charge-based repulsion (Figure S4D). Cryo-ET visualized this RNA exclusion at 30 min as abundant naked RNA molecules at the periphery of the amorphous condensed phase (Figure 3B; Video S3). Similar co-condensation and exclusion of RNA were observed for Hrp48 (Figure 3A). Therefore, an initial liquid state is essential for RNA incorporation *in vitro*.

Video S3. Tomographic reconstruction of Bruno-*oskar* 3'UTR condensate with labelled *oskar* 3'UTR added at 0 min (left), 30 min (middle) or 2 μM RFP-PTB added at 30 min (right), related to Figure 3 Scale bar 100 nm, each slice is 4 nm thick.

*oskar* granules *in vivo* show a distribution of sizes with a diameter of 400 ± 129 nm (Figure 1A). Condensates lack deterministic stoichiometry of the constituent molecules, resulting in assemblies of variable sizes that coarsen by coalescence and Ostwald ripening. Droplet growth is reduced when the constituents become physically trapped in an arrested state or when physical constraints of the intracellular space, such as cytoskeletal filaments, restrict droplet fusion (Feric and Brangwynne, 2013; Folkmann et al., 2021; Quiroz et al., 2020). Such mechanisms result in multiple microphases displaying a normal distribution of sizes (Ranganathan and Shakhnovich, 2020), as observed for *oskar* granules *in vivo* (Figure 1A). Quantification of our STED data revealed that an increase in granule volume correlated with higher RNA signal. However, there was no net increase in RNA concentration with increasing granule volume (Figure S4E). This confirms that the increase in RNA content toward the posterior pole, as previously suggested by diffraction-limited imaging (Little et al., 2015), is not due to dynamic partitioning of *oskar* RNA into the granules throughout the ooplasm. This finding highlights the potential importance *in vivo* of a short-lived liquid phase for RNA incorporation during granule assembly.

The observed non-dynamic nature of the condensates both *in vitro* and *in vivo* following aging raises questions about the incorporation of other proteins that associate with *oskar en route* to the posterior pole, such as PTB (Figure 2A). To test whether the solid condensates could incorporate proteins *in vitro*, we added mRFP-PTB to 30 min aged condensates of either Bruno or Hrp48 assembled with *oskar* 3′UTR. Although mRFP-PTB did not phase separate with *oskar* 3′UTR on its own (Figure 2C), it selectively partitioned into the condensates (Figure 3C). This enrichment was not only protein specific (mRFP-PTB versus TMR-BSA) but also RNA dependent, as Bruno or Hrp48-only condensates did not significantly concentrate PTB (Figure 3C). Cryo-ET revealed that PTB-enriched condensates were amorphous, indicating that PTB partitioning did not alter the molecular organization of the condensed state (Figure 3D; Video S3).

### Bruno PrLD plays a pivotal role in *oskar* granule assembly

**Figure S5:**
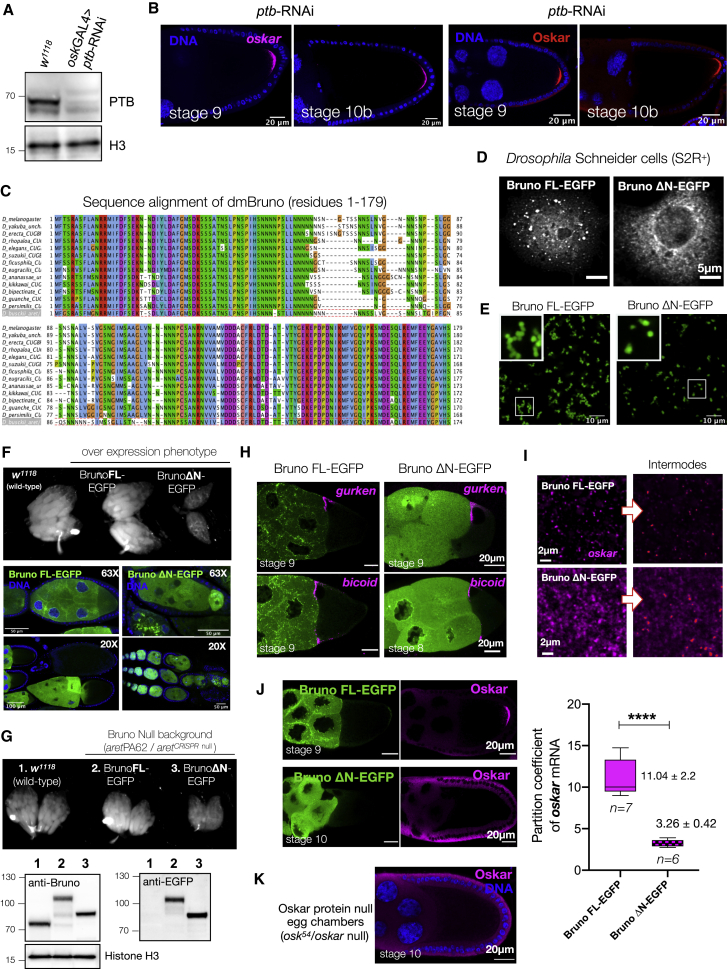
Role of Bruno and its PrLD in *oskar* function, related to Figure 4 (A) Western blot depicting knockdown of PTB upon RNAi driven by *oskar*GAL4 driver in the germline. (B) Posterior localization of *oskar* (detected by smFISH) and translation of Oskar protein are unaffected upon PTB knockdown. (C) Sequence alignment of amino acids 1–179 of *Drosophila melanogaster* Bruno and orthologs in other Drosophilids. (D) Expression of Bruno FL-EGFP and ΔN-EGFP in Schneider cells (S2R+). Note that Schneider cells do not express *oskar* mRNA. (E) *In vitro* reconstitution of 10 μM Bruno FL-EGFP and ΔN-EGFP in 150 mM NaCl buffer. (F) Overexpression of EGFP-tagged FL and ΔN Bruno in the germline by *oskar*GAL4 driver. Ovary morphology of the different genotypes shows the atrophic ovaries caused by overexpression of ΔN-EGFP. Protein is in green, and nuclei are stained with DAPI. (G) Morphology of ovaries of the indicated genotype is shown along with wild type. Western blot showing levels of expression of Bruno transgenes (in Bruno-deficient genetic background) with respect to wild type. After probing Bruno, the blot was stripped and re-probed with anti-EGFP antibody; histone H3 serves as loading control. Note that ΔN-EGFP expression levels cannot be directly compared with wild type or FL-EGFP, as in the case of ΔN-EGFP oogenesis is arrested, and the ovaries primarily contain younger-stage egg chambers. (H) Localization of *gurken* and *bicoid* is not affected upon expression of EGFP-tagged Bruno FL or ΔN in a Bruno-deficient background (*aret*PA62/*aret*^*CRISPR*^^null^). smFISH detected *gurken* (magenta) localizing correctly at the dorso-anterior corner and *bicoid* (magenta) at the anterior margin of the oocyte in mid-oogenesis. smFISH probe set used for *gurken* RNA (5′ to 3′): ggagctgctatatggcctg, ctacacacttgcatctccttg, tcggctcgaacaacaatctg, agcgtatgctctcggagaag, ctccaggcgattgagcaac, atcagtgattggtgtgctgc, tttcgggtgttgtcactgtc, tgaatctctgtctccttgtcg, tgttgttcaccatcggatgc, ggcaggaatggaagactgtg, agtcaccattccagctcttg, cgggaaaggagaagacgatg, gcgcaacgtaaagaaatatgg, tcgagtcgagtcccaatcc, gaacgcacacacacgaaac, gaccgattgtccaccactag, tctcctggatctgctgctg, caggtgtcggtactggatc, accgctctccatcgtagtc, agaacgtagagcgacgacag, ctgcttccggcgataatcc, tgcttatgcaggtgtagttg, tgccatccaacaaagaggag, aagcgaaacaaacgaaactaag, and for *bicoid* RNA (5′ to 3′): tggcaaaggagtgtggaaac, ctgaagctgcggatgttgg, tcgaagggatttcggaattg, ccatatcttcacctgggctg, gtccttgtgctgatccgat, ctccacccaagctaagagtc, gcgttgaatgactcgctgtag, tgtggcctccattgtagttg, ggtgattatggacctgctgc, gctggaagtcaaagtgatgg, gtagtacgagctgttgaagttg, gtgttaatggctcgtagacc, cacacagactcggactttcg, cttcttgctcgttccgtcg, cccttcaaaggctccaagatc, ctaaggctcttattccggtgc, ctccacgatttccggttcc, gcttgcattatcgtatccatcg, catccaggctaattgaagcag, atgaaactctctaacacgcctc, gtacaatcaggaacaacagtgg, acacggatcttaggactagacc, gaatagcgtattgcagggaaag, gcccaaatggcctcaaatg, ccgaaatgtgggacgataac. (I) Granular morphology of *oskar* RNPs is lost significantly in ΔN-EGFP. Representative single confocal plane of ooplasm with *oskar* (magenta) labeled by smFISH. Segmentation of granules from the dilute phase/cytoplasm was done using intensity-based segmentation and partition coefficient of *oskar* mRNA quantified. Error bars represent SD, and *n* denotes the number of oocytes analyzed. Unpaired Student’s t test were used for comparisons. Significance level: ^∗∗∗∗^ < 0.0001. (J and K) Oskar protein is not detected upon expression of ΔN. Immunostaining for Oskar protein (magenta) detected Oskar protein in Bruno FL, but not ΔN expressing egg chambers. In case of ΔN, signal (magenta) from the periphery of the egg chamber is background fluorescence (J) and is also detected in Oskar protein null flies (K).

Our *in vitro* reconstitutions show that Bruno and Hrp48 phase separate with *oskar* 3′UTR into liquid-like condensates that rapidly harden into a solid state, while PTB only partitions into preformed *oskar* 3′UTR-containing condensates. Moreover, *ptb*-RNAi in the germline had no visible effect on *oskar* granules and *oskar* function (Figures S5A and S5B). This led us to investigate how intrinsically phase separating Bruno and Hrp48 affect condensation and material properties of the granules *in vivo*. The early association and enrichment of Bruno with *oskar* in nurse cells and its role in higher-order particle formation indicate that Bruno may have a central role in granule assembly. However, manipulating Bruno levels in the germline is detrimental for oogenesis, preventing analysis of the effect of Bruno depletion on *oskar* granules (Filardo and Ephrussi, 2003; Webster et al., 1997).

**Figure 4:**
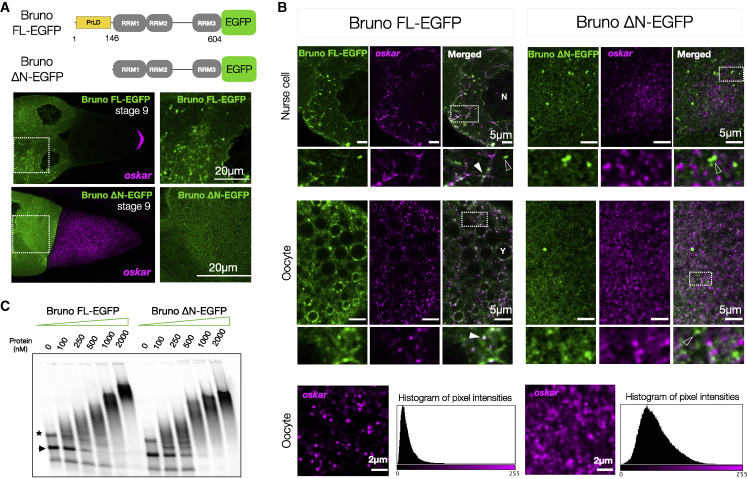
Bruno is essential for *oskar* granule assembly (A) Bruno constructs used for transgenesis. *oskar* RNA (magenta) smFISH in stage 9 egg chambers expressing Bruno FL- or ΔN-EGFP (green). (B) Single-plane confocal images of egg chambers expressing Bruno FL- or ΔN-EGFP (green) and *oskar* (magenta). White arrowheads: colocalization of protein with *oskar*; empty white arrowheads, protein puncta not associated with *oskar*; N, nurse cell nucleus; Y, yolk granule. Bottom: enlarged view of *oskar* granules (magenta) in ooplasm. Images acquired with independent microscope settings. A histogram of pixel intensities of the two images confirms the significant loss of granule formation and diffuse *oskar* RNA signal in Bruno ΔN-EGFP. (C) EMSA of *oskar* 3′UTR-atto633 (50 nM) with increasing concentrations of recombinant Bruno FL- and ΔN-EGFP. Arrowhead, *oskar* 3′UTR; ^∗^, dimeric form of the 3′UTR. See also Figure S5.

Analysis of Bruno primary sequence revealed that the N-terminal PrLD (Figure 2B) is highly conserved among Drosophilids (Figure S5C). A possible role of the N terminus in Bruno dimerization was reported (Kim et al., 2015). EGFP-tagged full-length Bruno (Bruno FL-EGFP) assembled into distinct granules in *Drosophila* Schneider cells, unlike the N-terminal truncated (Bruno ΔN-EGFP) version (Figure S5D). In contrast, both FL and ΔN proteins phase separated *in vitro* (Figure S5E). To unambiguously address the role of Bruno PrLD *in vivo*, where *oskar* RNA is present, we generated transgenic flies expressing Bruno FL- and ΔN-EGFP in a tissue-specific manner (Figure 4A). The transgenes were inserted by site-specific integration at an intergenic locus to avoid variability in expression due to chromosomal context. Overexpression of ΔN-EGFP was toxic to the germline and egg chambers degenerated at early stages (Figure S5F). Therefore, we expressed the transgenes in a Bruno-deficient background (*aret*PA62/*aret*^CRISPR null^). The FL protein rescued oogenesis, while expression of ΔN only partial rescued oogenesis, with egg chambers degenerating after stage 9 (Figure S5G). Notably, the FL protein formed distinct granules, many of which colocalized with *oskar* in the nurse cell cytoplasm, with enrichment on track-like structures (Figure 4B). ΔN-EGFP was largely diffuse, suggesting a role of the PrLD in granule formation (Figures 4A and 4B). Unlike FL-EGFP, expression of ΔN-EGFP led to complete failure in *oskar* localization at the posterior pole at stage 9 (Figure 4A). Localization of two other maternal RNAs, *gurken* and *bicoid*, was unaffected, confirming that the effect was specific for *oskar* (Figure S5H). Strikingly, *oskar* signal lost its granular appearance in the case of ΔN and instead appeared in diffuse puncta, indicating impairment of granule formation (Figure 4B). Quantification revealed a 3.5-fold reduction in *oskar* partitioning into granules upon ΔN expression compared with FL Bruno (Figure S5I).

We asked whether ΔN fails to bind *oskar* mRNA and therefore failed to localize with *oskar*. EMSA confirmed that recombinant ΔN, which retains all three RRMs of Bruno, is capable of binding *oskar* 3′UTR and forming higher-order oligomers (Figure 4C). This implies that ΔN likely binds *oskar in vivo* but fails to phase separate and form granules. Moreover, Oskar protein was not detected in ΔN egg chambers, unlike the FL (Figures S5J and S5K). Taken together, our experiments suggest that the scaffold protein Bruno, in particular its PrLD, plays a dominant role in *oskar* granule assembly. Given that granule assembly precedes hardening, the role of Bruno in hardening could not be assessed in the germline.

### PrLD of Hrp48 is crucial for *oskar* localization and translation

**Figure S6:**
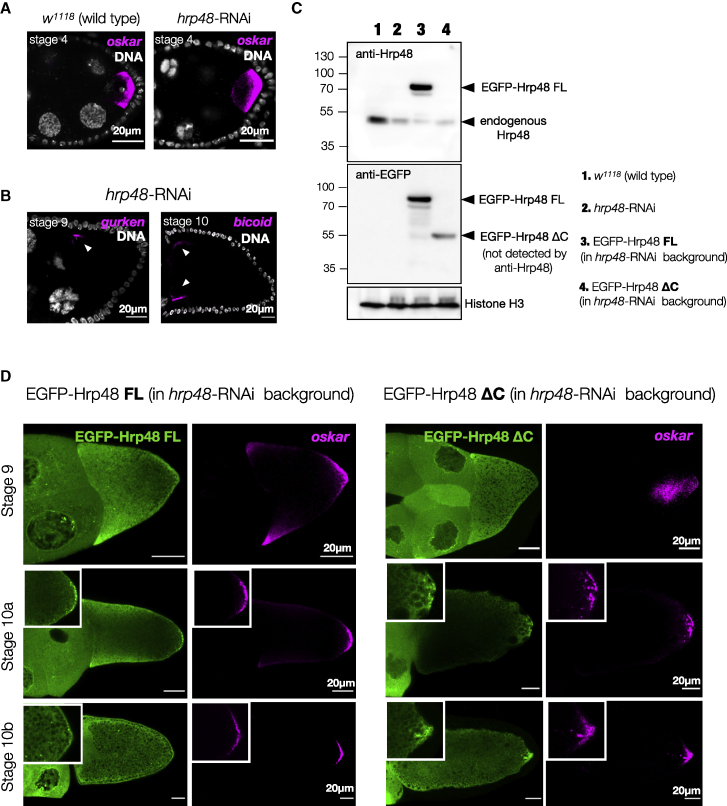
Role of Hrp48 and its PrLD in *oskar* localization, related to Figure 5 (A) *oskar* (magenta) enrichment in the oocyte is not affected by Hrp48 knockdown. (B) Localization of maternal RNAs *gurken* and *bicoid* (magenta) is not affected upon *hrp48*-RNAi. Maximum intensity projection of a Z volume of 5 μm. (C) Western blot of ovaries from flies of the indicated genotypes. The blot probed with anti-Hrp48 antibody has been stripped and re-probed with anti-GFP, as anti-Hrp48 failed to detect the truncated ΔC version. Histone H3 serves as loading control. (D) Representative confocal images of egg chambers of stages 9, 10a, and 10b shown with Hrp48 variants (green) and *oskar* (magenta) detected by smFISH from flies expressing the EGFP-tagged proteins in the *hrp48*-RNAi background. Insets show an enlarged version of the posterior pole.

**Figure 5:**
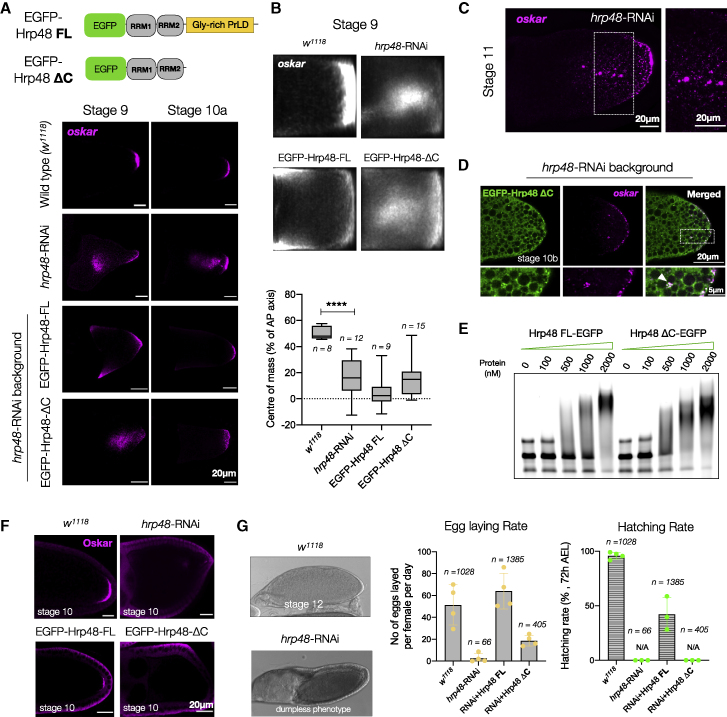
Loss of Hrp48 from the germline impairs *oskar* localization and translation (A) Hrp48 constructs used for transgenesis. *oskar* mRNA (magenta) smFISH in stage 9 and 10 egg chambers of the indicated genotypes. (B) Mean *oskar* RNA signal (grayscale) from stage 9 oocytes, anterior to the left. Position of the *oskar* center of mass relative to the geometric center of the oocyte (dotted horizontal line) along the anteroposterior (AP) axis. Error bars, SD; *n*, number of analyzed oocytes. Unpaired Student’s t test used for comparisons. Significance level: ^∗∗∗∗^ < 0.0001. (C) Clustering of *oskar* mRNA (magenta) into micron-sized condensates in *hrp48*-RNAi oocytes. (D) Confocal slice showing EGFP-Hrp48 ΔC (green) associates with *oskar* (magenta) granules (white arrowhead). (E) EMSA of *oskar* 3′UTR-atto633 (50 nM) with increasing concentrations of recombinant EGFP-Hrp48 FL and ΔC. (F) Immunostaining of Oskar protein (magenta); signal in follicle cells is background from the antibody also detectable in Oskar protein null egg chambers (Figure S5K). (G) Stage 12 *hrp48*-RNAi egg chamber showing the dumpless phenotype. Egg laying and hatching rate of flies of the indicated genotypes. Error bars: SD. See also Figure S6.

Unlike Bruno, Hrp48 knockdown in the germline does not cause early oogenesis arrest, allowing us to score the *oskar* phenotype. Enrichment of *oskar* in the oocyte was unaffected in *hrp48*-RNAi early-stage egg chambers (Figure S6A) (Huynh et al., 2004). Mislocalization was detected at stage 9 when *oskar* accumulated as a cloud in the center of the oocyte (Figures 5A and 5B). In later stages, larger assemblies of 2 to 4 μm in diameter were detected near the posterior pole, possibly arising from the coalescence of smaller granules (Figure 5C). Thus, Hrp48 knockdown did not abolish *oskar* granule formation or affect oocyte polarity (Figure S6B) but appeared to alter *oskar* granule behavior.

Mutations within the PrLD of Hrp48 were reported to impair *oskar* localization and translation but not binding to *oskar* mRNA (Huynh et al., 2004; Yano et al., 2004). To test the importance of the PrLD, we generated transgenic flies expressing EGFP-tagged FL or a PrLD-truncated version (ΔC) of Hrp48 (Figures 5A and S6C) and scored their ability to rescue the RNAi phenotype. At stage 9, the clustering of *oskar* granules in the center of the oocyte was not rescued by the expression of ΔC protein (Figure 5A). Expression of the FL protein rescued the central clustering, but anterior mislocalization was observed possibly due to higher expression levels of FL compared with wild-type Hrp48 (Figure S6C). At stage 10, posterior localization of *oskar* was rescued in FL oocytes, while the ΔC phenotype resembled that of the RNAi, with larger granules dispersed near the posterior pole (Figures 5A and S6D). The Hrp48 ΔC, which retains the two RRMs, localized with *oskar in vivo* (Figure 5D). EMSA confirmed that ΔC binds *oskar*, forming higher-order oligomers *in vitro* (Figure 5E). Translation of Oskar protein was impaired in the *hrp48*-RNAi flies, and egg laying was drastically reduced owing to the defects in nurse cell dumping (Huynh et al., 2004) (Figures 5F and 5G). Expression of FL rescued these phenotypes. Expression of ΔC failed to rescue translation (Figure 5F), egg laying was only partially rescued, and the eggs were unfertilized and failed to hatch (Figure 5G).

Depletion of Hrp48 from the germline or expression of ΔC did not abolish *oskar* granule formation, presumably owing to Bruno-driven LLPS. Instead, the granules appeared to coalesce into larger condensates. This suggests that *oskar* granules in Hrp48-depleted/truncated lines have altered physical properties that impair their native function and imply a role of the PrLD in modulating granule material properties. A similar observation was reported for Imp, a conserved component of *Drosophila* neuronal RNP granules (Vijayakumar et al., 2019). Biomolecular condensates exhibit a continuum of material and emergent properties that can be harnessed by the cell to specific needs (Alberti, 2017). Therefore, key questions arise as to the significance of the solid state in the physiological function of *oskar* in the germline.

### Manipulating the material properties of *oskar* granules impairs posterior localization

To address the importance of solidification following LLPS, we sought to drive the material properties of *oskar* granules toward a more liquid state without interfering with the endogenous scaffold proteins and the multitude of other functions they perform in the germline. We opted for tethering of an exogenous low complexity (LC) domain of human FUS, which reversibly phase separates into liquid condensates in living cells and *in vitro* (Burke et al., 2015; Shin et al., 2017). We genetically tethered the FUS LC domain to *oskar* mRNA, using the MCP-MS2 system.

**Figure 6:**
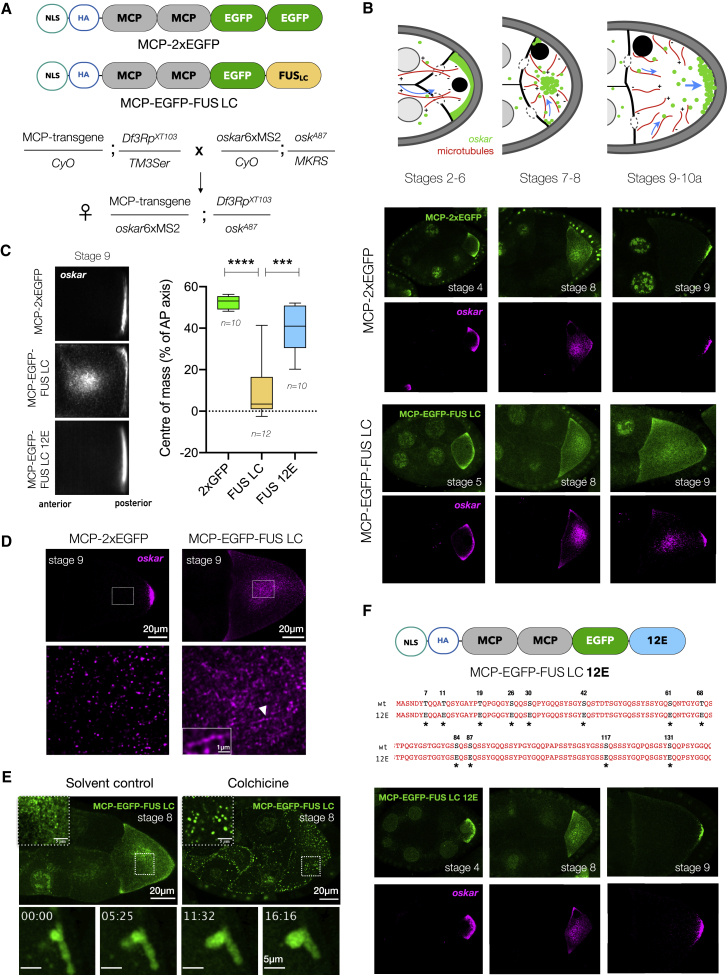
Manipulating the material properties of *oskar* granules affects RNA localization (A) Transgenic constructs and scheme of genetic crosses. NLS, nuclear localization signal; HA, hemagglutinin tag. (B) Cartoon representation of *oskar* RNA localization during oogenesis (adapted from Cha et al., 2002). Representative confocal images of *oskar* localization from early to mid-oogenesis upon 2xEGFP and FUS LC tethering; EGFP (green) and *oskar* (magenta). (C) Quantification of transport defects. Mean *oskar* RNA smFISH signal (grayscale); anterior to the left. Position of the *oskar* center of mass relative to the geometric center of the oocyte (dotted horizontal line) along the AP axis. Error bars, SD; *n*, the number of oocytes analyzed. Unpaired Student’s t test used for comparisons. Significance levels: ^∗∗∗^ < 0.001 and ^∗∗∗∗^ < 0.0001. (D) Stage 9 egg chambers with *oskar* RNA (magenta) smFISH; a central region in the oocyte (dotted white box) is enlarged below. White arrowhead, track-like structure with *oskar* granules. (E) Depolymerization of microtubules with colchicine in ovaries *ex vivo* (upper panel). Lower panel: liquid-like behavior of FUS LC-*oskar* granules upon colchicine treatment (min:sec). (F) MCP-EGFP-FUS LC 12E transgenic construct; ^∗^, 12 mutated residues. Representative images of early to mid-oogenesis stages; MCP-EGFP-FUS LC 12E (green) and *oskar* (magenta). See also Figure S7; Video S4.

**Figure S7:**
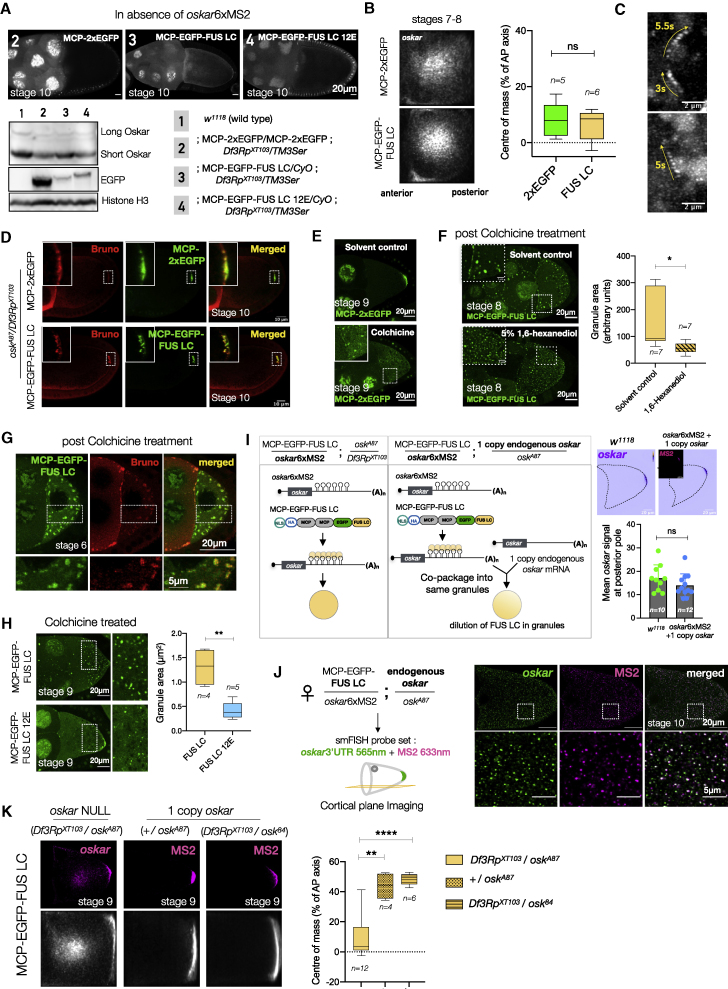
Manipulating the solid-like properties of *oskar* granules *in vivo*, related to Figure 6 (A) Representative images of stage 10 egg chambers with EGFP signal in grayscale; respective genotypes are indicated. Western blot of ovaries from the indicated genotypes shows transgene expression levels (anti-EGFP antibody) and Oskar protein. Histone H3 serves as a loading control. (B) Mean *oskar* RNA signal (grayscale) from smFISH data from stage 7–8 oocytes, anterior to the left. Position of the *oskar* center of mass relative to the geometric center of the oocyte (dotted horizontal line) along the AP axis is indicated. Error bars represent SD, and *n* denotes the number of oocytes analyzed. Unpaired Student’s t test were used for comparisons. NS, nonsignificant. (C) Maximum Z projections of selected regions of Video S4 (MCP-EGFP-FUS LC in grayscale) showing directed tracks marked with yellow arrows, and all three depicted particles having a velocity >0.5 μm/s. (D) Bruno association with *oskar* granules is not affected upon FUS LC tethering as revealed by immunostaining for Bruno protein. (E) Colchicine treatment of isolated ovaries in case of 2xEGFP tethering. (F) Treatment of egg chambers with 5% 1,6-hexanediol for 15 min after 2 h of colchicine treatment dissolves the large spherical assemblies partially. Quantification of granule size shows a significant reduction in 1,6-hexanediol treated samples. Error bars represent SD, and *n* denotes the number of oocytes analyzed. Unpaired Student’s t test were used for comparisons. Significance level: ^∗^ < 0.05. (G) Immunostaining of Bruno protein in egg chambers after colchicine treatment in the case of FUS LC tethering. (H) Colchicine treatment of stage 8–9 egg chambers induced the formation of large granules in case of FUS LC, which is significantly reduced in 12E. Error bars represent SD, and *n* denotes the number of oocytes analyzed. Unpaired Student’s t test were used for comparisons. Significance level: ^∗∗^ < 0.01. (I) Schematic representation of the rescue experiment in which one endogenous copy of *oskar* is supplied (left). Quantification of the mean *oskar* RNA signal (smFISH) from multiple stage 10a egg chambers of the indicated genotypes (right). *n* denotes the number of oocytes analyzed. Note that the *oskar*6xMS2 transgene is expressed from an *oskar* promoter. Unpaired Student’s t test were used for comparisons; ns, nonsignificant. (J) *oskar*6xMS2 mRNA and endogenous *oskar* transcripts co-package into the same granules. Two-color smFISH of egg chambers expressing one endogenous genomic *oskar* and the *oskar* 6xMS2 transgene with atto-565 probes against *oskar* (green) and atto-633 probes against MS2 loops (magenta). Bottom panels are enlarged from boxed regions. (K) Dilution of FUS LC per granule by an endogenous copy of *oskar* mRNA recuses the transport defects. *Oskar* distribution in representative stage 9 egg chambers detected by smFISH (magenta). Position of the *oskar* center of mass relative to the geometric center of the oocyte (dotted horizontal line) along the AP axis in indicated genetic backgrounds. *N* denotes the number of oocytes analyzed. Error bars represent SD. Unpaired Student’s t test were used for comparisons. Significance levels: ^∗∗^<0.01 and ^∗∗∗∗^<0.0001.

We generated transgenic flies expressing MCP-EGFP-FUS LC and a control line where the FUS LC is replaced by another EGFP molecule (Figure 6A) to ensure that the expressed proteins are of comparable size. Expression of these constructs in absence of MS2-tagged *oskar* had no effect on oogenesis and embryogenesis (Figure S7A). For genetic tethering, we crossed in an *oskar*6xMS2 transgene (Lin et al., 2008) that rescues oogenesis and embryogenesis in an *oskar* RNA-null genetic background (*osk*^*A87*^/*Df3Rp*^*XT103*^).

In early-stage egg chambers (stages 2–6), FUS LC-tethered *oskar* was transported from the nurse cells to the oocyte similar to the 2xEGFP-tethered control (Figure 6B). Therefore, FUS LC tethering did not impair *oskar* association with microtubules or the dynein machinery. At stages 7–8, the oocyte microtubule network reorganizes and *oskar* granules are transported by kinesin, initially away from the cortex to the interior, and eventually to the posterior pole at stage 9 (Cha et al., 2002). Movement of *oskar* granules toward the interior of the oocyte was indistinguishable between 2xEGFP and FUS LC tethering (Figures 6B and S7B). However, in stage 9 egg chambers, while the 2xEGFP-tethered *oskar* localized to the posterior pole, the FUS LC-tethered *oskar* was severely mislocalized: *oskar* appeared as a cloud in the center of the oocyte (Figures 6B and 6C), as observed upon Hrp48 knockdown/truncation (Figures 5A and 5B). Live imaging revealed directed runs of FUS LC-tethered *oskar* granules, indicating that microtubule association *per se* is not affected upon FUS LC tethering (Figure S7C; Video S4). Furthermore, the recruitment of Bruno as a primary granule scaffold was not affected upon FUS LC tethering (Figures S7D and S7G). We speculated that FUS-FUS interactions indeed resulted in altered material properties of the granules, provoking granule clustering in the oocyte center at stage 9. Careful examination of the cloud-like mass upon FUS LC tethering showed the occasional presence of larger granules and track-like segments with granules aligned like beads on a string (Figure 6D). We hypothesized that microtubule-directed transport of granules hinders their fusion into larger condensates. Depolymerization of microtubules in oocytes *ex vivo* by colchicine resulted in collapse of the diffraction-limited FUS-LC-tethered granules into large structures both in the oocyte and nurse cells that could fuse and relax like liquids and wet membrane surfaces, demonstrating the liquid properties of FUS LC-tethered *oskar* granules (Figures 6E and S7E; Video S5). 1,6-hexanediol partially dissolved the large granules (Figure S7F). Existence of small granules after dissolution indicated that the original solid phase persisted.

Video S4. Directed transport of FUS LC-tethered *oskar* granules (grayscale) near posterior pole, related to Figures 6 and S7

Video S5. Fusion of large FUS LC-tethered *oskar* granules (green) in oocyte upon colchicine treatment, related to Figure 6 A cropped region is presented in Figure 6E.

Phosphorylated and a phosphomimetic version of FUS LC abolish its phase separation *in vivo* and *in vitro* (Monahan et al., 2017; Rhoads et al., 2018; Shorter, 2017). We reasoned that if *oskar* localization defects arise from the induced liquid state of the granules, tethering an LLPS-deficient form of FUS LC should restore posterior localization. We generated transgenic flies expressing the phosphomimetic version of the LC (MCP-EGFP-FUS LC 12E). In contrast to wild-type FUS LC, the 12E version did not lead to aberrant central accumulation of *oskar* in stage 9 oocytes (Figures 6C, 6F, and S7H).

LLPS is also sensitive to the concentration and stoichiometry of condensate constituents. We tested whether reducing the amount of FUS LC in granules relative to the mRNA can rescue the mislocalization phenotype. To do so, in addition to the *oskar*6xMS2 transgene, we genetically provided an endogenous copy of *oskar*, whose transcripts cannot bind MCP-EGFP-FUS LC (Figure S7I). By virtue of 3′UTR mediated hitchhiking (Hachet and Ephrussi, 2004; Jambor et al., 2011), the *oskar*6xMS2 mRNA and endogenous *oskar* transcripts co-package into granules (Figure S7J). The altered FUS LC-to-mRNA stoichiometry reduced FUS LC phase separation resulting in the rescue of the localization defects (Figure S7K).

### An induced liquid state impairs *oskar* mRNA translation

**Figure 7:**
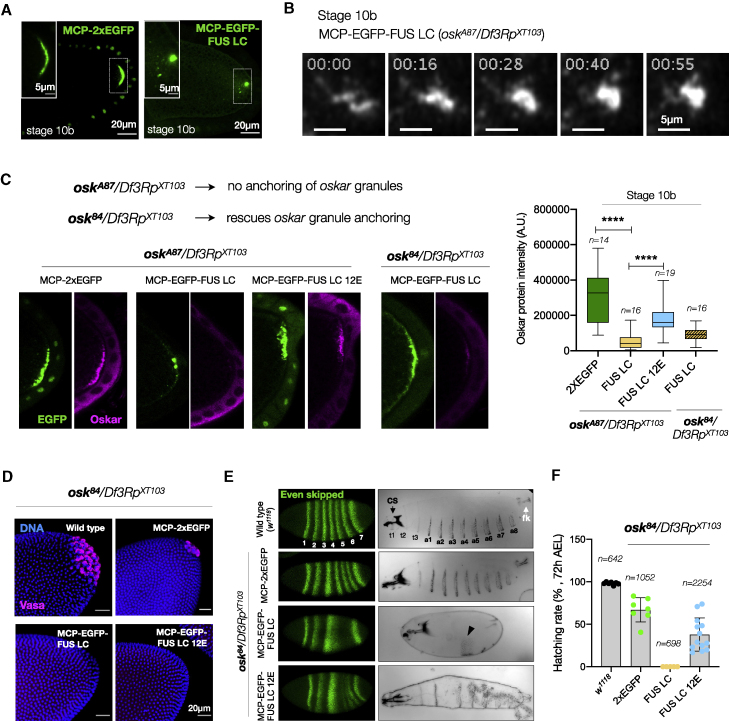
Manipulating the material properties of *oskar* granules interferes with *oskar* anchoring and translation and impairs embryonic development (A) Stage 10b egg chamber expressing MCP-2xEGFP or MCP-EGFP-FUS LC in *oskar* RNA-null background (*osk*^*A87*^/*Df3Rp*^*XT103*^). (B) Fusion of FUS LC-*oskar* granules (grayscale) at the posterior of stage 10b egg chambers. (C) Immunostaining and quantification of Oskar protein (magenta) intensity in *oskar* RNA-null stage 10b egg chambers. The genotype in the fourth panel is *osk*^*84*^/*Df3Rp*^*XT103*^ where the anchoring function is provided *in trans*. All images shown and quantified were acquired using identical microscope settings and representations contrast matched. Error bars, SD; *n*, number of analyzed oocytes. Unpaired Student’s t test used for comparisons. Significance level: ^∗∗∗∗^ < 0.0001. (D) Pole cells identified by Vasa immunostaining (magenta) and nuclei stained with DAPI (blue) in embryos of the indicated genotypes. (E) Pattern of even skipped (Eve) stripes (green) and representative cuticles of embryos of the indicated genotypes; anterior to the left, ventral to the bottom. t1–t3, thoracic segments; a1–a8, abdominal segments; cs, head skeleton (black arrow); fk, filzkörper (white arrow); black arrowhead, patchy band of denticles. Refer to Figure S8H for phenotypic classes observed. (F) Quantification of hatching rate of eggs of the indicated genotypes. Error bars, SD; *n*, number of eggs scored. See also Figure S8; Video S6.

In late oogenesis, ooplasmic streaming (starting stage 10b) ensures localization of *oskar* granules by facilitated diffusion and anchoring (Becalska and Gavis, 2009; Glotzer et al., 1997; Palacios and St Johnston, 2002; Theurkauf, 1994). Whereas a posterior crescent of *oskar* mRNA was present in the control 2xEGFP, posterior accumulation FUS LC-*oskar* granules led to the formation of spherical, micron-sized, and dynamic condensates (Figures 7A and 7B; Video S6) as observed upon loss of Hrp48 (Figure 5C). The large condensates were loosely anchored at the pole and subsequently detached into the ooplasm.

Video S6. Fusion of FUS LC-tethered *oskar* granules (grayscale) into larger condensates at the posterior pole, related to Figure 7

**Figure S8:**
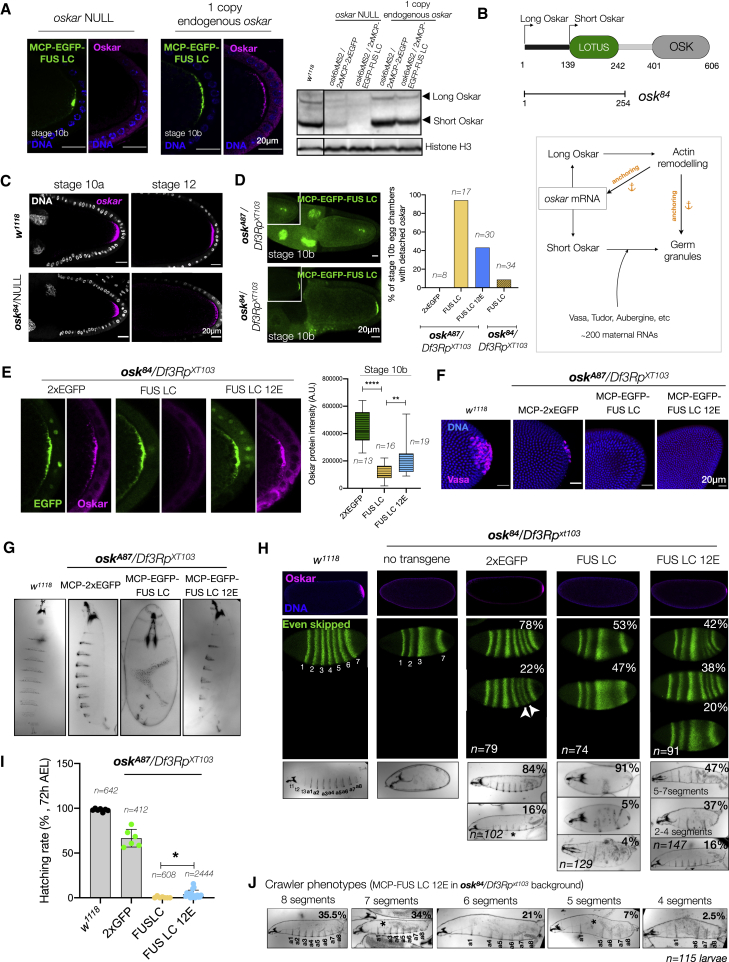
Effect of altered physical state of *oskar* granules on embryonic development, related to Figure 7 (A) Oskar protein immunostaining in oocytes and western blot confirms loss of translation upon FUS LC tethering in *oskar* null background and rescue of translation in presence of an endogenous copy of *oskar*. Arrows mark the two isoforms of Oskar protein. Note that the reduction in Oskar protein levels in case of MCP-2xEGFP compared with wild type is due to the *oskar*-RNA-null (*osk*^*A87*^/*Df3Rp*^*XT103*^) background of the flies. The black line after lane 1 indicates that lane 1 is not immediately adjacent to the other lanes in the original blot. (B) Schematic representation of Oskar protein domain architecture indicating the start sites of the long and short isoforms. Nonsense mutant *osk*^*84*^ encodes 254 residues from the N terminus and provides the anchoring function. Flowchart representation of multiple interdependent functions of Oskar protein isoforms in actin remodeling, anchoring, and organization of the germ plasm (adapted from Tanaka and Nakamura, 2011). (C) Anchoring of *oskar* RNPs is rescued in females expressing *osk*^*84*^ allele; NULL indicates the other chromosome: *oskar*-CRISPR-RNA-null allele. smFISH for *oskar* mRNA (magenta) on egg chambers of the indicated genotypes shows *oskar* anchoring in stage 10 (left) and stage 12 (right) egg chambers. (D) Anchoring defects are rescued in *osk*^*84*^/*Df3Rp*^*XT103*^ background. Representative images of stage 10b egg chambers expressing MCP-EGFP-FUS LC (green) in the indicated genetic backgrounds. Quantification of *oskar* detachment phenotype from images of stage 10b egg chambers expressing the indicated transgenes in absence or presence of anchoring provided *in trans*. *n* denotes the number of egg chambers analyzed. (E) Immunostaining of egg chambers for Oskar protein (magenta) upon provision of anchoring *in trans* by the *osk*^*84*^ allele. The EGFP signal in green confirms the rescue of anchoring. All images shown (and used for quantification) were acquired using identical microscope settings and representations are contrast matched. Quantification of the Oskar signal intensity from the posterior of several egg chambers confirms the reduction of translation upon FUS LC tethering and partial translation using the FUS 12E construct. Note that the FUS LC panel is also shown in Figure 7C. Error bars represent SD, and *n* denotes number of analyzed oocytes. Unpaired Student’s t test were used for comparisons. Significance levels: ^∗∗^ < 0.01 and ^∗∗∗∗^ < 0.0001. (F) Formation of the germline is impaired upon Fus LC and 12E tethering in an *oskar* RNA-null background (*osk*^*A87*^/*Df3Rp*^*XT103*^). Reduction of pole cell numbers is noted in 2xEGFP tethering compared with wild type. Pole cells at the posterior of embryos at nuclear cycle 14 are identified by Vasa (magenta) immunostaining. Nuclei stained with DAPI (blue). (G) Representative cuticles of embryos reveal severe patterning defects upon FUS LC tethering. Anterior faces the top and ventral to the left. (H) Immunostaining of Oskar protein (magenta) in early embryos, Eve (green) stripe patterns in cellular blastoderm embryos, and cuticle phenotypes are shown for the indicated genotypes. Representative images of the major phenotypic class observed for each genotype are shown in Figure 7E. *n* denotes the number of embryos or cuticles analyzed. (I) Quantification of the hatching rates of eggs from females expressing the indicated transgene in an *oskar* RNA-null background (*osk*^*A87*^/*Df3Rp*^*XT103*^). Number of eggs scored per genotype is depicted in the graph. Note that data for *w*^*1118*^ are also shown in Figure 7F. Error bars represent SD, and *n* denotes the number of analyzed eggs. Unpaired Student’s t test were used for comparisons. Significance level: ^∗^ < 0.05. (J) Cuticle analysis in the case of MCP-EGFP-FUS LC 12E (in *osk*^84^/*Df3Rp*^*XT103*^ background) by collecting only those specimens present in the yeast paste placed in the center of the agar plate, to which the viable and crawling larvae are attracted. The larvae were then classified based on the number of segments. Majority of the crawlers had six to eight abdominal segments. ^∗^ denotes incomplete segments.

*oskar* mRNA is translationally repressed during transport. Derepression, by yet poorly understood mechanisms, upon localization results in Oskar protein production at the posterior pole. Similar to the *hrp48*-RNAi phenotype, Oskar protein was barely detected upon FUS LC tethering (Figure 7C). In contrast, the 12E construct only partially impaired Oskar translation (Figure 7C). Notably, the translation phenotype could also be suppressed by providing an additional copy of endogenous *oskar* RNA (Figure S8A).

Western blotting confirmed the reduction in levels of both the long and short Oskar isoforms (Figure S8A). Short Oskar induces assembly of the pole plasm (Ephrussi and Lehmann, 1992), and the long isoform organizes the posterior cortex of the oocyte and anchors the pole plasm (Vanzo et al., 2007; Vanzo and Ephrussi, 2002) (Figure S8B). This made us question whether the observed detachment of FUS-LC-tethered *oskar* granules is a consequence of the loss of anchoring due to reduced Oskar protein levels, resulting in further reduction of *oskar* mRNA translation.

The N terminus of Oskar is sufficient for anchoring (Hurd et al., 2016; Vanzo and Ephrussi, 2002). To uncouple the interdependency between anchoring and translation, we provided the anchoring function *in trans* using an *oskar* nonsense mutant allele, *osk*^*84*^, which encodes the N-terminal 254 residues and completely rescued anchoring at stages 9–10, with minor delocalization only in late stages (Vanzo and Ephrussi, 2002) (Figures S8B and S8C). *oskar* detachment observed in 90% of stage 10b egg chambers upon FUS LC tethering was rescued when anchoring was provided, and the granules did not collapse into large condensates (Figure S8D). However, *oskar* translation was still compromised by FUS LC tethering (Figures 7C and S8E). Thus, the liquid-like state induced by FUS LC tethering, rather than defective anchoring, is responsible for the observed translational shutdown.

### Altered material state of *oskar* granules is detrimental to embryonic development

A localized source of Oskar is crucial for germline development and patterning of the embryo (Lehmann and Nusslein-Volhard, 1986). Whereas a small amount of Oskar is sufficient for patterning the abdomen, a high local concentration is required to induce germ cell formation (Ephrussi and Lehmann, 1992; Smith et al., 1992). To investigate the effect of changed material properties of *oskar* granules on germline formation, we assessed the pole cell formation in embryos at the blastoderm stage. While wild-type (*w*^*1118*^) embryos had an average of 25–30 pole cells, 2xEGFP tethering in an *oskar* RNA-null background resulted in 10–12 pole cells. FUS LC tethering led to the complete absence of pole cells, whether or not anchoring was provided *in trans*. Although Oskar was detected in FUS LC 12E expressing oocytes, the amount of protein was not sufficient to induce pole cell formation in either *osk*^*A87*^ or *osk*^*84*^ genetic backgrounds (Figures 7D and S8F).

We also examined anteroposterior patterning by analyzing the expression of pair-rule gene *even skipped* (*eve*) and segmentation in embryos. Tethering of 2xEGFP resulted in the formation of all seven Eve stripes and consequently eight abdominal segments (a1–a8) in the majority of embryos, as in wild type (Figures 7E, S8G, and S8H), and no head defects were observed. Upon FUS LC tethering, the embryos displayed a loss of abdominal segmentation in both *osk*^*A87*^ or *osk*^*84*^ backgrounds, as reported for strong loss-of-function *oskar* alleles (Lehmann and Nusslein-Volhard, 1986). The loss of Eve stripes 4, 5, and 6 and abdominal segments is a consequence of loss of Oskar protein as observed for *osk*^*84*^*/Df3Rp*^*XT103*^ embryos (Figure S8H), confirming that the FUS LC phenotype stems from reduced Oskar protein production. 66% of the 2xEGFP embryos hatched, while FUS LC embryos did not hatch at all, whether in an *osk*^*A87*^ or *osk*^*84*^ backgrounds (Figures 7F and S8I). Expression of FUS LC 12E resulted in a spectrum of embryo phenotypes, from wild type to loss of some Eve stripes and abdominal segments, as a consequence of heterogeneity in Oskar protein levels. Remarkably, in the *osk*^*84*^*/Df3Rp*^*XT103*^ background, 44% of the FUS LC 12E embryos hatched, with the majority of the larvae exhibiting 5–7 abdominal segments (Figures 7F and S8J). This underlines how modulation of the physical state of *oskar* granules toward a more liquid phase impacts the development of the future embryo.

## Discussion

Our study of *oskar* transport granules in the *Drosophila* oocyte elucidates key principles of granule assembly and reveals the importance of regulation of condensate properties for asymmetric expression of a maternal RNA that induces embryonic patterning and germline formation.

Stereospecific molecular interactions seed RNA-protein complexes, which through further multivalent interactions and molecular crowding *in vivo* form mesoscopic assemblies with emergent properties. *oskar* RNA assemblies appear dimmer in nurse cells than in oocytes (Figure S4B). It was previously shown that the majority of nurse cell *oskar* RNA assemblies correspond to 1- or 2-copy *oskar* mRNA particles, while granules in the oocyte are of higher RNA copy number (Little et al., 2015). Consistent with its role in granule assembly, the primary scaffold protein Bruno associates strongly with *oskar* in nurse cells, and the RNA and protein are co-detected on microtubule-like tracks (Figure 4B). We speculate that in addition to single RNA molecules, some nurse cell puncta represent RNP granule precursors in the form of small clusters as has been described for the stress granule scaffold protein G3BP1 (Guillen-Boixet et al., 2020) and engineered condensates (Shimobayashi et al., 2021). Entry into the heavily crowded ooplasm and recruitment of additional proteins may promote condensation of these precursors into granules containing multiple *oskar* mRNA molecules. Scaffold proteins Bruno and Hrp48 are classic examples of RBPs with a modular architecture: a disordered PrLD and structured RNA-binding domains. RRM-driven sequence-specific binding to *oskar* ensures the selection of specific mRNA, and PrLD-driven LLPS promotes granule assembly via self-association as well as multivalent interactions with other proteins bound to the mRNA. We verified that in absence of their PrLDs, both Bruno and Hrp48 can bind *oskar* and promote the formation of higher-order oligomers (Figures 4C and 5E), but functions pertaining to *in vivo* granule formation and granule material properties are affected. *oskar* mRNA was shown to dimerize by kissing-loop interactions (Hachet and Ephrussi, 2004; Jambor et al., 2011). This raises the question whether the mRNA has an architectural role in granule assembly (Yamazaki et al., 2019; Jain and Vale, 2017; Ferrandon et al., 1997; Paillart et al., 1996; Van Treeck and Parker, 2018; Van Treeck et al., 2018). However, our *in vitro* reconstitutions indicate that self-assembling scaffold proteins promote condensation of *oskar* 3′UTR under conditions in which the 3′UTR alone does not condense into visible assemblies. Condensation can in turn promote the formation and stabilization of RNA-RNA interactions (Figure 2E).

The minimal *oskar* RNP condensates rapidly mature *in vitro* into a non-dynamic state with respect to fusion or molecular exchange. The liquid state appears to be essential for RNA incorporation, as solidification precludes RNA entry into the condensates. Functional biomolecular condensates exhibit a spectrum of material properties. While the nucleolus, stress granules, and P granules are liquid-like, Balbiani bodies and PCM are more solid-like (Audas et al., 2016; Boke et al., 2016; Brangwynne et al., 2009; Lafontaine et al., 2021; Patel et al., 2015; Schmidt and Görlich, 2015; Woodruff et al., 2017, 2018). *oskar* granules constitute transport cargoes that travel distances up to 100 μm to localize. Artificially inducing a long-lived liquid-like state drastically compromised the localization efficiency of *oskar* (Figure 6C). Therefore, it is plausible that hardening through non-covalent cross-linking of scaffold proteins and RNA confers mechanical stability that endows *oskar* granules with properties that support long-distance transport (Figure 2E). In a large, polarized cell such as the developing oocyte, dynamic microtubule network organization is essential for the transport of maternal RNAs, proteins, and organelles. While cytoskeletal filaments can act as platforms that promote condensation by increasing local concentrations (Hernández-Vega et al., 2017; Wiegand and Hyman, 2020), viscoelastic filaments can also restrict condensate dynamics and fusion. The coalescence of FUS LC-tethered *oskar* granules (Figure 6E) upon depolymerizing the microtubule network suggests that active transport on microtubule tracks prevents fusion of the small granules into larger ones. Cytoskeleton-driven spatial segregation of condensates has been observed in the *Xenopus laevis* oocyte nucleus, where a nuclear actin network prevents sinking and fusion of nucleoli (Feric and Brangwynne, 2013). Furthermore, once localized to the posterior, the persistent presence of *oskar* RNA is toxic to pole cells, and it is actively segregated from the germ granules at the posterior of the oocyte and embryo (Eichler et al., 2020; Little et al., 2015). Retaining or reverting to a liquid state of *oskar* granules is therefore a potential threat to this segregation, as co-condensation of *oskar* granules and germ granules could result in co-packaging of *oskar* RNA and other pole cell-destined maternal transcripts with detrimental consequences to the embryo.

Condensates *in vivo* are multicomponent systems that contain a complex mixture of macromolecules, where the relative stoichiometry of the component molecules determines condensate architecture and material properties (Boeynaems et al., 2019) or condensate function (Case et al., 2019). Both Bruno and Hrp48 form solid-like condensates with *oskar* RNA *in vitro* (Figure 2). *In vivo*, Bruno seeds *oskar* granules (Figure 4), and Hrp48 recruitment is important to maintain their solid-like properties (Figure 5). Therefore, both proteins and possibly their relative stoichiometries act to determine the final material state of the granules. RNA-protein stoichiometry also plays a role in determining material properties: adding one copy of endogenous *oskar* reduces the protein-to-mRNA ratio inside the FUS LC-tethered granules, resulting in reduced FUS LC phase separation, reversion to a solid-like phase, and restoration of *oskar* granule function (Figure S7).

We further show that although minimal *oskar* condensates rapidly develop into non-dynamic assemblies, they selectively enrich client RNA-binding protein PTB (Figure 3C). For a large mRNA such as *oskar* (3 kb), Bruno binding to specific sites in the 3′UTR nucleates higher-order oligomers, presumably by crosslinking the 3′UTRs, thus forming networks of associative Bruno “stickers” and largely protein-free RNA segments acting as “spacers” (Guillen-Boixet et al., 2020; Wang et al., 2018). Such network-like architecture of the scaffold would favor partitioning of client proteins in an RNA-dependent manner depending on available valencies (binding sites) on the mRNA. In this way, Hrp48 partitioning maintains the material properties of the granules (Figure 5), while PTB recruitment confers an additional layer of translation regulation (Besse et al., 2009). Thus, the first layer of selectivity can arise from relative RNA-binding affinities of proteins, as well as the availability of binding sites. Another layer of selectivity presumably arises from the porosity of the condensate. Condensate assembly involves multivalent, cooperative interactions among the constituent macromolecules, which form a physically cross-linked network. The degree of cross-linking and further molecular rearrangements determine the final material state, from viscous liquids to viscoelastic solids to amyloid fibers (Holehouse and Pappu, 2018). Imaging of the solid-like minimal *oskar* condensates at molecular resolution with cryo-ET revealed an amorphous appearance, confirming that the initial liquid phase hardens into a glassy solid, adding to accumulating evidence that liquid-to-solid transitions on a short timescale result from entanglement rather than restructuring into amyloids (Saha et al., 2016; Woodruff et al., 2017). Glasses are easy to fluidize and hence can be advantageous to cells not only to shut down biochemical reactions but also to rapidly respond to changes in condensate composition and physical factors such as temperature, pH, etc. (Alberti and Hyman, 2021; Jawerth et al., 2020). A lack of structural reorganization into stable amyloids in the solid state might facilitate translational activation of *oskar* granules at the posterior pole. Our experiments show that a 100-kDa protein such as RFP-PTB can partition into the hardened condensates, while the ∼330-kDa *oskar* 3′UTR itself is excluded. Therefore, it is expected that small globular proteins can enrich in an RNA-dependent manner, but megadalton complexes such as ribosomes are excluded, ensuring translation repression. Other possible client proteins are Staufen, which associates with *oskar* only once the RNA enters the oocyte (Little et al., 2015) and is involved in dynein-to-kinesin motor switching (Gáspár et al., 2021), as well as putative translation de-repressors (Castagnetti and Ephrussi, 2003; Chang et al., 1999; Dold et al., 2020; Micklem et al., 2000; Wilson et al., 1996), including RNA helicases (Nakamura et al., 2001). It is possible that granule remodeling by partitioning of client proteins, without affecting material properties, is required for translation activation. Transition of *oskar* granules into a solid provides a mechanism to ensure selective partitioning of *oskar* mRNA, proper localization, and regulated translation that are key to the development of oocyte and embryo.

### Limitations of the study

Our findings highlight the importance of physiological liquid-to-solid phase transition of *oskar* granule for their *in vivo* function, yet some outstanding questions remain. It is unclear how the physical state of RNP granules mechanistically regulates their translation status. Liquid-like condensates have been assigned dynamic biochemical functions, while solid-like condensates are conceived as dormant sites of storage. Therefore, translational impairment upon imparting a long-lived liquid-like state on *oskar* granules seems counterintuitive. Nevertheless, there is evidence of translation inhibition in liquid-like condensates formed by the LC domain of fragile X mental retardation protein (Tsang et al., 2019). Conversely, solid-like nuclear amyloid bodies were shown to be hubs of local nuclear translation under stress conditions (Theodoridis et al., 2021). We have not observed fusion or dissolution of solid *oskar* granules at the posterior in wild-type late-stage oocytes, indicating that reversion to a liquid state is not required to initiate translation in the granules (Figure S1). Mechanistic understanding of translation de-repression of *oskar* mRNA is limited, preventing us from exploring the FUS LC-induced translational shutdown. The liquid-like state induced by FUS LC tethering possibly interferes with the remodeling of the localized granules. The N-terminal domain of Bruno has been reported to be phosphorylated by protein kinase A (PKA) *in vitro* (Kim et al., 2015). A constitutively active PKA mutant has been shown to induce ectopic translation of Oskar protein (Yoshida et al., 2004). However, *in vivo* targets of PKA have not been identified. PKA-driven phosphorylation of scaffold proteins might remodel condensate architecture and initiate *oskar* translation. An altered physical state might interfere with such a mechanism.

## STAR★Methods

### Key resources table

**Table:** 

REAGENT/RESOURCE	SOURCE	IDENTIFIER
**Antibodies**		

Rabbit anti-Oskar (1:3000; WB & IF)	In-house	N/A
Rabbit anti-Hrp48 (1:2000; WB)	In-house	N/A
Rabbit anti-Bruno (1:1000; WB & 1:3000 IF)	In-house	N/A
Rabbit anti-PTB (1:2000; WB)	In-house	N/A
Rabbit anti-EGFP (1:5000; WB)	Torrey Pines Biolabs	Cat# TP401; RRID:AB_10890443
Rabbit anti-Histone H3 (1:2500; WB)	Abcam	Cat# ab1791; RRID:AB_302613
Rat anti-Vasa (1:500; IF)	In-house	N/A
Mouse anti-Even-skipped (1:500; IF)	Gift of Justin Crocker	∗DSHB 2B8

**Chemicals**		

1,6-Hexanediol	Sigma	Cat# 240117
Colchicine	Sigma	Cat# C9754
Insulin	Sigma	Cat# I9278
Fetal Bovine Serum (FBS)	Life Technologies	Cat# 10082147
Schneider’s Drosophila medium	Life Technologies	Cat# 21720024
SumoStar protease	Life Sensors	Cat# 4110
PEG-4000	Thermo Scientific	Cat# EL0011
ATP	Thermo Scientific	Cat# R0441
RNase A	Thermo Scientific	Cat# EN0531
Benzonase	Sigma	Cat# E1014
Tetramethyl Rhodamine BSA	Thermo Scientific	Cat# A23016
Shandon ImmuMount	Thermo Scientific	Cat# 9990402

**Critical commercial assays**		

Effectene Transfection reagent	Qiagen	Cat# 301425
X-tremeGENE HP DNA Transfection reagent	Roche	Cat# 06 366 236 001
MEGAscript T7 transcription kit	Thermo Scientific	Cat# AMB13345
StrepTrap HP	Merck	Cat# GE28-9075-47
HisTrap HP	Merck	Cat# GE17-5248-01
HiLoad 16/600 Superdex 200pg	Merck	Cat# GE28-9893-35

**Plasmids**		

pFastBAC 1-mRFP-PTB	This study	N/A
pCoofy63-BrunoFL-EGFP	This study	N/A
pCoofy63-BrunoΔN -EGFP	This study	N/A
pCoofy63-Hrp48FL-EGFP	This study	N/A
pCoofy63-Hrp48ΔC -EGFP	This study	N/A
UASp-attB-K10-Bruno FL-EGFP	This study	N/A
UASp-attB-K10-Bruno ΔN-EGFP	This study	N/A
pU6-BbsI-chiRNA	Gratz et al. (2013)	Addgene Cat# 45946
pHD-scarless dsRED	Kate O'Connor-Giles	Addgene Cat# 64703
UASp-attB-K10-EGFP-Hrp48FL	This study	N/A
UASp-attB-K10-EGFP-Hrp48ΔC	This study	N/A
∗pHsp83-NLS-HA-tdMCP-2xEGFP	This study	N/A
Dendra2-FUS WT	Patel et al. (2015)	N/A
∗pHsp83-NLS-HA-tdMCP-EGFP-FUS LC	This study	N/A
MBP-FUS FL-12E	Monahan et al. (2017)	Addgene Cat# 98652
∗pHsp83-NLS-HA-tdMCP-EGFP-FUS LC 12E	This study	N/A
pActin5C-Bruno FL-EGFP	This study	N/A
pActin5C-Bruno ΔN -EGFP	This study	N/A
Oligonucleotide probes smFISH probes are listed in supplementary figure legends S2 (*oskar*) and S5 (*gurken*, *bicoid*)	Gáspár et al., (2017b)	N/A

**Fly strains (D. melanogaster)**		

*w*^*1118*^	∗BDSC	BDSC Cat# 3605; RRID:BDSC_3605
*oskar6xMS2*/*CyO*	Lin et al. (2008)	N/A
*oskar6xMS2:MCP-EGFP/Tm3Sb*	Lin et al. (2008)	N/A
*mRFP-Nup107*	Hampoelz et al. (2019)	N/A
w[1118]; PBac{y[+mDint2] GFP[E.3xP3]=vas-Cas9}VK00027	BDSC	BDSC Cat# 51324; RRID:BDSC_51324
y[1] M{vas-int.Dm}ZH-2A w[∗]; PBac{y[+]-attP-3B}VK00033	BDSC	BDSC Cat# 24871; RRID:BDSC_24871
vas-phi-ZH2A, PBac{y[+]-attP-9A}VK00018	BDSC	BDSC Cat# 9736; RRID:BDSC_9736
*aret*CRISPR-dsRED/*CyO*	This study	N/A
*If*/*CyO*; UASp-BrunoFL-EGFP/*Tm3ser*	This study	N/A
*If*/*CyO*; UASp-BrunoΔN-EGFP/*Tm3ser*	This study	N/A
*bruno*-RNAi : *P{TRiP.HMC02374}attP2*	BDSC	BDSC Cat# 44483; RRID:BDSC_51324
*hrp48*-RNAi : *P{TRiP.JF01478}attP2*	BDSC	BDSC Cat# 31685; RRID:BDSC_51324
*ptb*-RNAi : *P{TRiP.GLV21034}attP2*	BDSC	BDSC Cat# 35669; RRID:BDSC_51324
*If*/*CyO*; UASp- EGFP-Hrp48FL- /*Tm3ser*	This study	N/A
*If*/*CyO*; UASp- EGFP-Hrp48ΔC- /*Tm3ser*	This study	N/A
*PTB-EGFP trap* : *w-;Bl/CyO;GFP-PTB/TM6b*	Besse et al. (2009)	N/A
*Bruno-EGFP trap* : *w-; GFP-Bruno/CyO*	BDSC	BDSC Cat# 60144; RRID:BDSC_51324
pHsp83-MCP-2xEGFP/*CyO*	This study	N/A
pHsp83-MCP-EGFP-FUS LC/*CyO*	This study	N/A
*If*/*CyO*; pHsp83-MCP-EGFP-FUS LC 12E/*Tm3ser*	This study	N/A
*oskar*GAL4/*Tm3sb*	BDSC	BDSC Cat# 44242; RRID:BDSC_51324
*oskar*^*A87*^	Jenny et al. (2006)	N/A
*oskar*^*attP,3P3GFP*^	Gáspár et al., (2017a)	N/A
*oskar*^*84*^	Lehmann and Nusslein-Volhard (1986)	N/A
*Df3Rp*^*XT103*^	Lehmann and Nusslein-Volhard (1986)	N/A

**Software**		

Fiji	Schindelin et al. (2012)	https://fiji.sc
xsPT Fiji plugin	Gaspar and Ephrussi (2017)	https://github.com/Xaft/xs/blob/master/_xs.jar
Cort Analysis Fiji plugin	Gaspar et al. (2014)	N/A
Imaris	Bitplane	https://imaris.oxinst.com
Huygens Essential	Scientific Volume Imaging, Hilversum, the Netherlands	https://svi.nl/Huygens-Deconvolution
FRAP Analyser	Halavatyi and Terjung (2017)	https://github.com/ssgpers/FRAPAnalalyser
PLAAC	Lancaster et al. (2014)	http://plaac.wi.mit.edu/
IUPred	Mészáros et al., (2018)	https://iupred2a.elte.hu
IMOD v.4.9	Kremer et al. (1996)	https://bio3d.colorado.edu/imod/
TOM package	Nickell et al. (2005)	MATLAB (MathWorks)
SerialEM v3.7.2	Mastronarde (2005)	https://bio3d.colorado.edu/SerialEM/
WARP	Tegunov and Cramer (2019)	http://www.warpem.com/warp/
Prism 8.0	GraphPad	https://www.graphpad.com/
JalView 2.11.1.3	Waterhouse et al. (2009)	http://www.jalview.org
BioRender.com	Graphical Abstract created with BioRender.com	https://biorender.com

∗ td: tandem, BDSC: Bloomington Drosophila Stock Center, DSHB: Developmental Studies Hybridoma Bank.

### Resource availability

#### Lead contact

Further information and requests for resources and reagents should be directed to and will be fulfilled by the Lead contact Anne Ephrussi (anne.ephrussi@embl.org).

#### Materials availability

All unique materials and reagents generated in this study are available from the Lead contact with a completed material transfer agreement.

### Experimental model and subject details

*Drosophila melanogaster* stocks were maintained at 25°C on standard cornmeal agar. 3-6 day old female flies with typically half as many male flies were transferred to vials with fresh yeast 24 h before experiments.

Details are described in the methods detail section.

### Method details

#### Fly stocks

The fly strains used in the study are listed in the Key Resources Table.

#### Generation of transgenic flies

##### *P*-element mediated germline transformation

Transgenic flies expressing MCP-2xEGFP/MCP-EGFP-FUS LC/MCP-EGFP-FUS LC 12E were generated by P-element transformation. Briefly, 2xEGFP, FUS LC (gift from Tony Hyman) and FUS LC 12E (Addgene) were cloned downstream of the Hsp83 promoter and P-element transgenesis performed in *w*^*1118*^ flies using standard procedures (Rubin and Spradling, 1982). After eye-color based screening for positives using the truncated *mini-white* marker gene, transgenes were balanced on the respective chromosomes (*CyO* or *Tm3Ser* balancers) to establish stable stocks. For FUS LC 12E, inserted on chromosome III, recombination with an *oskar* CRISPR null allele expressing EGFP from the 3xP3 promoter (*oskar*^*attP,3P3GFP*^ ; (Gáspár et al., 2017a)) was carried out.

##### Bruno CRISPR knock out flies

CRISPR null flies were generated as described in FlyCripsr (https://flycrispr.org). Two guide RNAs (gRNA), one in exon 1 and another in exon 2 of the *aret* gene, were designed (5′gRNA-CGGAGAAAUCGAAAAUCAUG and 3′gRNA-CGGCGAGAAGGAACCGGAUC) and cloned into an empty pU6-gRNA vector independently. Homology arms of 1kb each were cloned into the pHD-scarless dsRED donor vector. A mixture of gRNA plasmid and donor plasmid was injected into *w*^*1118*^ embryos; *PBac{y[*+mDint*2]=vas-Cas9}VK00027* embryos. dsRED positive flies were selected and balanced with *CyO* and used as an *aret* knock-out line.

#### PhiC31 integrase-mediated site-specific insertion

The CDS of Bruno FL (1-604) and ΔN (147-604) were tagged at the C-terminus with mEGFP using the Gateway cloning system (Invitrogen) in an intermediate pActin5c vector. Subsequently the EGFP-tagged sequence was sub-cloned into the UASp-*attB* vector and injected into VK-33 (y*[1] M{vas-int.Dm}ZH-2A w[^∗^]; PBac{y[+]-attP-3B}VK00033*) embryos for site-specific insertion into the *attP* landing site on chromosome III. Selection for positive transformants was based on eye color (*mini-white* gene). The transgene was balanced with *Tm3Ser* and the *PhiC31* integrase was crossed out to obtain the final stocks. For Hrp48 truncations, EGFP tagging was done at the N terminus of FL and ΔC (1-205) using the Gateway cloning system (Invitrogen) in a pActin5c vector and then sub-cloned into the UASp-*attB* vector. VK-18 (*vas-phi-ZH2A, PBac{y[+]-attP-9A}VK00018*) embryos were injected for site-specific insertion into the *attP* landing site on chromosome II. The transgenes were balanced with *CyO*.

#### Live imaging of egg chambers

Ovaries of the desired genotype were dissected and mounted onto glass-bottom dishes in a 20 μl drop of Schneider’s medium (with 10% fetal calf serum (FBS) and 200 μg/ml insulin) with an adjacent drop of Voltalef 10S oil. Individual egg chambers of desired stages were pulled under the oil with fine tungsten needles under a stereo microscope. Live imaging was done on a Zeiss LSM880 Airy Scan microscope (Airy Fast mode) with 40X/1.1 NA water immersion objective at room temperature with a pixel size of 50 nm x 50 nm at a frame rate of 1-2 fps.

#### *Ex vivo* treatments of whole ovaries

##### 1,6-Hexanediol

Ovaries of the desired genotype were dissected in PBS and incubated in Schneider’s medium (with 10% FBS and 200 μg/ml insulin) with 5% 1,6-HD for 15 min at RT on a nutator. Water was used as a solvent control.

##### Colchicine

Ovaries of the desired genotype were dissected in PBS and incubated in Schneider’s medium (with 10% FBS and 200 μg/ml insulin) with 100 μg/ml Colchicine for 2h at RT on a nutator. 100% ethanol was used as a solvent control. Live imaging was done after 90 min of colchicine treatment (100 μg/ml RT). Colchicine was present in the imaging medium.

##### Protein expression in insect cells

All plasmids are listed in the Key Resources Table. pFastBAC1 and pFastBAC-based pCoofy63 vectors were used for cloning. pCoofy63 vector has a N-terminal 6xHis-SumoStar tag and a C-terminal Twin-Strep tag. Recombinant proteins Bruno, Hrp48 and PTB were expressed and purified from insect cells (Sf-21) using the baculovirus expression system to mimic the close-to-physiological state of the proteins. For generation of recombinant bacmid, pFastBAC1 or pFastBAC1-based pCoofy63 shuttle vector was transformed into DH10EmBacY *E. coli* competent cells by electroporation, followed by blue-white screening to select for recombinant bacmid. This was followed by bacmid isolation and PCR-based verification. Sf-21 cells grown at a density of 0.5-1.0 x 10^6^ cells/ml were transfected with recombinant bacmid using X-tremeGENE HP DNA transfection reagent (Roche) and V_0_ harvested after 72h of transfection. Virus amplification was carried out by infecting 25 ml Sf-21 cells with V_0_ and V_1_ harvested one day post proliferation arrest. Usually 1 L of uninfected Sf-21 cells (0.5-0.7 x 10^6^ cell/ml) were infected with V_1_ at a ratio of 1:100 and cells were harvested 72 h after infection. Harvested cells were flash-frozen in liquid N_2_ and stored at -80°C.

##### Protein purification

###### Bruno-EGFP, Bruno ΔN-EGFP, Hrp48-EGFP, & Hrp48 ΔC-EGFP

Sf-21 cells expressing recombinant proteins were resuspended in lysis buffer (20 mM Tris-HCl pH 7.5, 500 mM NaCl, 1 mM EDTA supplemented with 0.01% TritonX-100, 1x tablet of Complete Mini Protease Inhibitor cocktail (Roche), 2 mM MgCl_2_, Benzonase (Sigma)) for 10 min on ice for digestion of RNA/DNA by Benzonase nuclease, followed by lysis using a microfluidizer. Lysate was centrifuged at 16000 x g at 4°C for 20 min to remove debris.

Proteins were C-terminally tagged with TwinStrep tag and affinity purified from the clarified lysate using a 5ml StrepTrap HP column. The N-terminal 6xHis-Sumostar solubility tag was maintained during purification. Briefly, the clarified lysate was injected into the column, the bound proteins washed in 5-6 column volumes wash buffer (20 mM Tris-HCl pH 7.5, 500 mM NaCl, 1 mM EDTA) and finally eluted in Elution buffer (2.5 mM desthiobiotin (Sigma) in 20 mM Tris-HCl pH 7.5, 500 mM NaCl, 1 mM EDTA). Fractions were analyzed by SDS-PAGE and desired fractions pooled and dialysed overnight at 4°C using a 12-14 kDa MWCO membrane (Spectrapor) in dialysis buffer (20 mM Tris-HCl pH 7.5, 500 mM NaCl) to remove EDTA and desthiobiotin. Post dialysis, the protein was concentrated to 5 ml and subjected to high resolution size exclusion chromatography in storage buffer (20 mM Tris-HCl pH 7.5, 300 mM NaCl, 2m M MgCl_2_, 5% glycerol, 0.5 mM TCEP) using HiLoad 16/600 Superdex 200pg column. Fractions were analyzed by SDS-PAGE and desired fractions pooled and concentrated using a concentrator with 50 kDa MWCO (Amicon). Droplet formation was checked during the purification to ensure that phase separation or aggregation did not occur during the concentration. Aliquots were flash-frozen and stored at -80°C.

##### mRFP-PTB

mRFP-PTB was affinity purified by Ni-NTA chromatography. Briefly, Sf-21 cells were resuspended in Lysis buffer (20 mM Tris-HCl pH 7.5, 500 mM NaCl, 5% glycerol, 40 mM imidazole, supplemented with 0.01% TritonX-100, 1x tablet of Complete Mini Protease Inhibitor cocktail (Roche), 2 mM MgCl_2_, Benzonase (Sigma)) for 10 min on ice for digestion of RNA/DNA by Benzonase, followed by lysis using a microfluidizer. Lysate was centrifuged at 16000 x g at 4°C for 20 min to remove debris. The clarified lysate was injected into a HisTrap HP column 5 ml, bound fractions were washed with 3-5 column volumes of wash buffer (20 mM Tris-HCl pH 7.5, 500 mM NaCl, 5% glycerol, 40 mM Imidazole) and eluted using a 40-600 mM gradient of Imidazole. Fractions were analyzed by SDS-PAGE, desired fractions pooled and dialyzed overnight at 4°C using a 12–14 kDa MWCO membrane (SpectraPor) in wash buffer to remove imidazole along with TEV protease (produced by the EMBL Protein Expression and Purification Core Facility) to cleave off the 6xHis tag. The untagged protein was separated from the 6xHis-tagged proteins and cleaved tags by a second round of Ni-NTA chromatography, and the flow through (containing tag-cleaved protein) was collected, concentrated and subjected to high resolution size exclusion chromatography in storage buffer (20 mM Tris-HCl pH 7.5, 300 mM NaCl, 2 mM MgCl_2_, 5% glycerol, 0.5 mM TCEP) using HiLoad 16/600 Superdex 200pg column. Fractions were analyzed by SDS-PAGE and desired fractions pooled and concentrated using a concentrator with 50 kDa MWCO (Amicon MERCK). Droplet formation was checked to ensure that phase separation or aggregation did not occur during the concentration steps. Aliquots were flash-frozen and stored at -80°C.

All purifications were done using an Akta FPLC system (GE Life sciences). Protein extinction coefficients were calculated using ProtParam (Expasy) and concentrations were measured with diluted samples at 280 nm in a NanoDrop (Thermo Scientific).

#### *In vitro* transcription and fluorescent labelling of transcripts

*In vitro* transcription (IVT) was performed using a MEGAscript T7 transcription kit (Invitrogen) according to the manufacturer’s instructions. Briefly, template for IVT was prepared by PCR using T7-forward primer and gene specific reverse primers. 200 ng template DNA was used for a 20 μl transcription reaction for 2-3 h at 37°C; template DNA was digested with Turbo DNase and RNA was precipitated with lithium chloride (LiCl) and dissolved in ultrapure water (Invitrogen). For fluorescent labelling, the transcription reaction was spiked with 5-amino-allyl UTP (Biotium) at 1:4 (amino allylUTP: UTP). Fluorescent labelling was carried out with 3-fold molar excess of atto633 NHS-ester (Atto-Tec GmbH) in 0.1 M NaHCO_3_ at RT for 2 h, protected from light. RNA was precipitated at -20°C with absolute ethanol and sodium acetate, pH 5.5, centrifuged at 16000x g 15 min 4°C, followed by 2 washes with ice-cold 70% ethanol. RNA was dissolved in ultrapure water (Invitrogen). Integrity of the unlabeled and labelled transcripts was verified with SYBR Safe stain (473 nm), as well as by fluorescent gel imaging (635 nm) in a Typhoon biomolecular imager.

#### *In vitro* phase separation assays

All *in vitro* phase separation experiments were carried out in assay buffer (20 mM Tris-HCl pH 7.5, 150 mM NaCl, 2 mM MgCl_2_, 5% glycerol, 0.5 mM TCEP). No crowding agent was included, unless mentioned in figure descriptions. Protein concentrations were adjusted so that the final salt concentration was 150 mM NaCl in the assay.

Frozen aliquots of proteins were thawed and centrifuged at maximum speed to clear aggregates. For Bruno-EGFP and Hrp48-EGFP, the 6xHis-SumoStar tag was cleaved during the phase separation reaction using 1 U SumoStar protease (Life Sensors) per 20 μl reaction for 30 min at RT. *oskar* 3’UTR RNA (labelled or unlabeled) was added to the assay where specified. Details of individual experiments are indicated in respective figure schematics and legends. Reactions with all the components were assembled in Eppendorf tubes and immediately spotted on 96-well non-binding μclear plates (Greiner Bio-one), incubated up to 30 min for enzymatic tag removal and imaged using a Leica SP8 confocal microscope. For *in vitro* condensate ageing assays, the reactions were incubated in Eppendorf tubes for 30 min, followed by addition of RNA/proteins and then spotted onto 96-well plates for imaging. In the case of 30 min time point, condensates were imaged 15 min after fluorescently labelled RNA was added, to allow enough time for the droplets to settle down on the glass surface.

#### Electrophoretic Mobility Shift Assay (EMSA)

EMSA was carried out as described previously (Besse et al., 2009). 50 nM of atto-633-labelled *oskar* 3’UTR (labeled as described above) was incubated with increasing concentrations of indicated proteins for 20 min at RT in assay buffer (20 mM Tris-HCl pH 7.5, 150 mM NaCl, 2 mM MgCl_2_, 5% glycerol, 0.5 mM TCEP). The reactions were resolved on a 0.8% agarose gel in 0.5X TBE run at 100V constant current at 4°C. Fluorescent gel imaging (635 nm) was performed in a Typhoon biomolecular imager.

#### Single molecule Fluorescent in situ hybridization (smFISH)

The protocol used for smFISH has been described in detail in Gaspar et al. (2017b).

##### Labelling of NH_2_-ddUTP

Atto633-NHS-ester or atto565-NHS-ester (Atto-Tec GmbH) was reconstituted with anhydrous DMSO in a desiccation chamber to 20 mM final concentration. Conjugation of Amino-11-ddUTP (NH2-ddUTP, Lumiprobe) with NHS-esters was done using a 2-fold molar excess of dye–NHS-ester in the presence of 0.1 M NaHCO3 pH 8.3 for 2 h at RT protected from light. The reaction was quenched by adding 1 M Tris HCl pH 7.4 to 10 mM final concentration and the concentration adjusted to 5 mM with nuclease free water.

##### Probe labelling

Probe sequences are described in the Key Resources Table. Non-overlapping DNA oligos (Sigma) 18–22 nt long were selected using the smFISHprobe_finder.R script (Gaspar et al., 2018) and reconstituted to 250 μM with nuclease-free water. An equimolar mixture of a probe set was enzymatically conjugated to fluorescently labelled ddUTP (atto633 or atto565) using Terminal Deoxynucleotidyl Transferase (Thermo Scientific). Labelled oligos were precipitated with absolute ethanol, sodium acetate pH 5.5 and linear acrylamide and reconstituted with nuclease free water.

##### Hybridization

Freshly dissected ovaries were immediately fixed in 2% paraformaldehyde (PFA) in PBS with 0.05% TritonX-100 at RT for 20 min. The fixed ovaries were rinsed once and washed for 10 min with PBS containing 0.1% TritonX-100, followed by prehybridization at 42°C for 10 min with shaking in hybridization buffer (300 mM NaCl, 30 mM sodium citrate pH 7.0, 15% (v/v) ethylene carbonate, 1 mM EDTA, 50 μg/mL heparin, 100 μg/mL salmon testes DNA, 1% Triton X-100). 50 μL of prewarmed probe mixture (2.5 nM per individual oligonucleotide) was added and hybridization performed for 2-3h at 42°C. Excess probe was washed with 2 washes with hybridization buffer at 42°C for 20 min each, followed by a final wash in PBS containing 0.1% TritonX-100. Ovaries were mounted in mounting medium (80% glycerol, 2% propyl gallate).

#### Immunostaining and western blotting

For immunostaining, freshly dissected ovaries were fixed in 4% PFA in PBS for 20 min, extracted in permeabilization buffer (1% TritonX-100 in PBS) and blocked in blocking buffer (0.5% BSA, 0.3% TritonX-100 in PBS) for 1h. For embryo collections, virgin female flies were mated with double the number of wild-type (*w*^*1118*^) males with a drop of yeast paste in apple juice agar plates for 2–3 days at 25°C before egg collection. For Eve staining, 2-4 hour old eggs were collected and dechorionated using 50% bleach followed by extensive washing with distilled water. The embryos were fixed at the interface of 4% paraformaldehyde and heptane for 20 min at RT in glass vials. This was followed by devitellinization by vigorous shaking in a 1:1 mix of heptane and methanol. Fixed embryos were stored in 100% methanol at -20°C. Before staining the embryos were rehydrated in wash buffer PBT (0.1% TritonX-100 in PBS) followed by blocking with Blocking Reagent (Roche) in PBT. Ovaries were blocked using 0.5% BSA in PBT. Incubation with primary antibodies was carried out overnight at 4°C followed by three washes in PBT 15 min each. Incubation with Alexa Fluor-secondary antibodies diluted in 10% goat serum in PBS was carried out for 2 h at RT, followed by 3 washes in wash buffer for 15 min each. Nuclei were stained with DAPI (1:2500 in wash buffer). Samples were mounted in mounting media (80% glycerol, 2% propyl gallate).

For western blotting, an equal number of freshly dissected ovaries of desired genotypes was directly resuspended and lysed in 1× Laemmli buffer (Invitrogen) supplemented with 5% β-mercaptoethanol. After boiling at 95°C for 10 min, samples were centrifuged at maximum speed to remove debris and equal volumes loaded for SDS-PAGE analysis in 4-12% NuPAGE pre-cast gels (Invitrogen). Following wet transfer to PVDF membrane (Millipore) for 2 h at 4°C, the membrane was blocked in 5% skimmed milk in TBST (TBS-0.1% Tween-20), incubated with primary antibody overnight at 4°C followed by 3 washes in TBST 10 min each. Incubation with a secondary antibody was done at RT for 1h followed by 3 washes and detection by chemiluminescence (BioRad).

#### Pole cell staining

10-15 virgin females of the desired genotype were mated with double the number of *w*^*1118*^ males and maintained on yeast paste for 2-3 days at 25°C before egg collection. 2-4 h old eggs were collected on apple juice agar plates, dechorionated with 50% bleach for 2 min, and fixed in preheated fixation buffer (0.4% NaCl, 0.3% Triton X-100 in PBS) at 92°C for 30s. This was immediately followed by devitellinization by vigorous shaking in a 1:1 mix of heptane and methanol. Fixed embryos were stored in 100% methanol at −20°C. For pole cell staining, fixed embryos of the desired genotype from multiple collections were pooled, washed 3–5 times with 0.1% Triton X-100 in PBS, blocked with blocking buffer (0.5% BSA, 0.3% TritonX-100 in PBS) and incubated with anti-Vasa (1:500) primary antibody overnight at 4°C. This was followed by 3 washes in wash buffer, incubation with secondary antibody at RT for 1h. The nuclei were stained with nuclear stain 4′,6-diamidino-2-phenylindole (DAPI).

#### Embryonic cuticle preparations

10-15 virgin females of the desired genotype were mated with double the number of *w*^*1118*^ males while being fed with yeast paste for 2-3 days at 25°C. Prior to egg collection, flies were placed in cages and allowed to lay eggs overnight on apple juice agar plates. Next morning the plates were collected and the eggs aged for 24 h at 25°C. After collection, the eggs were dechorionated with 50% bleach for 2 min, washed extensively with water and transferred to glass slides. Excess water was removed and the samples were mounted in Hoyer’s medium and Lactic acid (Sigma), covered with a cover slip and baked overnight at 65°C. Imaging was performed on a bright field microscope.

#### Transfection of S2R+ cells

*Drosophila* Schneider cells (S2R+) were cultured in Schneider’s Drosophila medium (Life Technologies) containing 10% FBS (Life Technologies) and 1% Penicillin-Streptomycin. Transient transfection of cells was carried out using Effectene Transfection reagent (Qiagen) according to the manufacturer's protocol. Cells were transfected with 200 ng plasmid in 24 well plates. After 24 hours cells were re-plated on Concanavalin A-coated coverslips for 1 hour prior to fixation with 4% PFA. Post-fixation, cover slips were washed with 0.1% Triton X-100 containing PBS, mounted using Shandon ImmuMount (Thermo Scientific) and imaged with a water immersion objective.

#### Image acquisition

##### Confocal microscopy

For high resolution image acquisitions of egg chambers, laser scanning confocal microscopy was carried out using a Leica TCS SP8 microscope with a HC PL APO 63x/1.30 Glycerol CORR CS2 glycerol-immersion objective. Images were acquired in the Leica Lightning mode with adaptive deconvolution. Low resolution imaging was performed with a HC PL APO 20X/0.75 CS2 Air objective. For *in vitro* droplet experiments, images were acquired with an HC PL APO 40x/1.10 W CORR CS2 water-immersion objective without subsequent deconvolution.

##### Stimulated emission depletion super resolution microscopy

3D STED microscopy was performed on a Leica SP8 STED 3X microscope equipped with a HC PL APO CS2 93X/1.30 Glycerol-immersion objective. Egg chambers were mounted in 80% glycerol containing mounting medium. Atto-633 was excited at 633 nm with a white-light laser (WLL) and STED was performed at 775 nm. 30% STED depletion beam power was used for image acquisition. Images were collected in line averaging mode (16 lines) and the pinhole was set to 1.0 Airy units. For XZ-scan, a pixel size of 40 nm x 12 nm was used. 3D volumes were acquired with a voxel size of 40 nm x 40 nm x 60 nm. Acquired STED images were deconvolved with Huygens Professional (SVI, version 20.10) prior to analysis.

#### Fluorescence recovery after photobleaching

For Fluorescence Recovery After Photobleaching (FRAP) recordings, *in vitro* reconstituted condensates (assembled with Bruno-EGFP /Hrp48-EGFP/hFUS-EGFP) were excited with the 488 nm line of a white light laser on a Leica SP8 laser scanning confocal using a HC PL APO 40x/1.10 W CORR CS2 water-immersion objective. A circular region of interest of 1.4 -1.5 μm^2^ was selected within the condensate and bleaching was carried out with 100% laser power of the same laser line. Three images were acquired prior to bleaching, following which fluorescence intensity was recorded for up to 55 sec at a frame interval of 0.35 sec (Figure S3H) and up to 3 min at a frame interval of 2 sec (Figures 2D and S3I).

#### Quantification of absolute protein concentrations in granules *in vivo*

For calculating absolute concentrations per granule, Bruno and PTB EGFP-trap fly lines tagged with EGFP at the endogenous locus were used. A calibration curve was generated using bacterially expressed recombinant EGFP. Freshly dissected ovaries were fixed with 4% PFA for 15 min followed by washes with PBS containing 0.1% Triton X-100. Fixed egg chambers were separated in recombinant EGFP storage buffer (20 mM Tris-HCl pH 7.5, 150 mM NaCl, 2 mM MgCl_2_, 5% glycerol, 0.5 mM TCEP) in 96-well non-binding μclear plates (Greiner Bio-one) and imaged with a Leica SP8 confocal microscope using a 40X HC PL APO 40x/1.10 W CORR CS2 water-immersion objective. Imaging was done in a cortical plane to resolve individual granules near the posterior cortex. A range of recombinant EGFP concentrations was imaged under identical optical settings at identical height from the cover slip surface. For calculations, single granules were marked with a ROI and concentrations estimated from a calibration curve of EGFP intensities.

#### Quantification of *oskar* RNA concentrations in granules *in vivo*

*oskar* smFISH was carried out on wild-type (*w*^*1118*^) ovaries and egg chambers were imaged with a Leica SP8 using a HC PL APO 63x/1.30 Glycerol CORR CS2 glycerol-immersion objective and deconvolved using Leica Lightning adaptive deconvolution. Cortical plane acquisition was performed with detectors in photon-counting mode. Assemblies in the nurse cells appear much dimmer than those in oocytes, and as previously shown correspond to 1 or 2-copy *oskar* mRNA, while granules in the oocyte are much brighter with higher RNA copy number (Little et al., 2015). Imaging of the nurse cell and oocyte cytoplasm in the same field of view was therefore challenging and imaging settings were carefully balanced to avoid saturation effects. Particles were segmented in xsPT Fiji plugin and intensities quantified. mRNA copy number was calculated by fitting multiple Gaussian functions to the corresponding signal intensity distributions taken from the nurse cells using the normalmixEM procedure of the mixtools package in *R* studio (Gáspár et al., 2021; Gaspar et al., 2017b). The μ value of Gaussian fit that described the largest portion of the distribution in the nurse cells was taken as the signal intensity of a unit (the intensity of a single mRNA molecule). The volume of granules as obtained from 3D STED volume imaging was then used to calculate absolute mRNA concentration per granule.

#### Cryo-electron tomography

##### Screening condensates on TEM grids

All assay conditions were checked with light microscopy on a standard non-binding surface. Prior to vitrification, samples were also screened on EM grids to ensure stability of condensates on the grid material. Quantifoil R2/1 Cu 200 mesh holey Carbon grids were glow-discharged for 45 s. 4 μl of sample was spotted on the grid, incubated for 1 min, followed by spiking of glutaraldehyde (fixative) to a final concentration of 0.05% and imaged with a Zeiss Axiovert wide-field microscope after 30 s.

##### Vitrification by plunge freezing

2After assessing condensate stability on grids, identical conditions were used for sample spotting on the grids and proceeded to plunge-freezing. Grids were blotted from both sides for 2 s with 0 blot force, followed by a drain time of 2 s and immediately plunged into liquid ethane at liquid nitrogen temperature using a Vitrobot Mark 4 (FEI Company/Thermo Fisher Scientific, Eindhoven, Netherlands) with the chamber set at 22°C, 90% humidity. The frozen grids were stored in liquid nitrogen until further processing.

##### Cryo-electron tomography

Cryo-electron tomography was carried out on a 300 kV (FEI Company/Thermo Fisher Scientific, Eindhoven, Netherlands) Titan Krios microscope equipped with a field-emission gun, a Quantum post-column energy filter (Gatan, Pleasanton, CA, USA), a K2 direct detector camera (Gatan) and a Volta phase plate (FEI Company/Thermo Fisher Scientific, Eindhoven, Netherlands). Data recording was done in dose fractionation mode using SerialEM software v3.7.2 (Mastronarde, 2005). Tilt series were collected using a dose symmetric scheme (Hagen et al., 2017) and a Volta phase plate (Danev et al., 2014) in nano-probe mode, pixel size at the specimen level of 2.12 Å, 3–4 μm defocus, tilt increment of 2° at a constant dose of 2.1 e^-^/ Å ^2^ for all tilts. Motion correction and Contrast transfer function (CTF) estimation was performed using WARP (Tegunov and Cramer, 2019). Tilt series alignment with patch-tracking and tomographic reconstructions were done using the IMOD software package, version 4.9.4 (Kremer et al., 1996). Aligned images were binned to the final pixel size of 8.51 Å. For tomographic reconstruction by back-projection, the radial filter options were set at default values (cut off, 0.35; fall off, 0.05). The reconstructed tomograms were filtered with a Gaussian filter of radius of 3 pixels using the TOM package implemented in Matlab (Mathworks).

### Quantification and statistical analysis

#### Image analysis

For all image representations in the figures, Fiji was used. Maximum intensity Z-projections were also made with Fiji. Fluorescence time-lapse movies were generated with Fiji and custom arrows incorporated using the plugin developed in Daetwyler et al. (2020). Intensity-based thresholding, histogram generation & intensity line profile was done using Fiji. For intensity line profiles in Figure 3A, single confocal plane images were used and intensity values along a line drawn were obtained from Fiji and plotted using Prism 8. For histogram generation in Figure 4B, a single confocal plane image was used and pixel histogram obtained in Fiji. For calculation of granule size in Figure S7, intensity-based thresholding was carried out for the granules and area of the segmented particles were obtained by particle analysis in Fiji.

#### Analysis of STED data

STED data for Figure 1A was prepared in Fiji. Image stacks acquired with 3D STED were analyzed using Imaris (BitPlane, version 9.5.1). Granules were first segmented and the shape parameters of the segmented particles were obtained from Imaris. Granule volumes and signal intensities are plotted in Figure S4E. Calculation of aspect ratios was performed in Fiji. Line profiles along the major and minor axis of a granule were generated to calculate the lengths of the major and minor axes, respectively. The Aspect Ratio was then calculated as the ratio of the Major axis to the Minor axis. 3D STED can achieve near isotropic resolution dependent on the STED depletion laser power used. To assess the effect of STED depletion beam power on the apparent size of the object we measured the aspect ratio of 500 nm microspheres (Tetraspeck™ beads) under identical imaging conditions. Beads were diluted in PBS and embedded in mounting medium containing 80% glycerol (as used for oocytes). Image stacks were collected in line averaging mode (16 lines) and the pinhole was set to 1.0 Airy units. Using 10, 30, 50 and 100% of 775 nm laser power we obtained aspect ratios of 1.53, 1.23, 1.03 and 0.91. Therefore, with increasing STED depletion power, the ideal bead object approaches an aspect ratio of ∼1, while at lower STED depletion power, ratios above 1 are obtained. *oskar* granules *in vivo* were acquired using 30% beam power (see above) and displayed mean aspect ratio of ∼1.4. This ratio is in good agreement with the ratio 1.23 obtained from the perfectly spherical beads, confirming that *oskar* granules are spherical in shape.

#### Colocalization analysis

Frequency of colocalization of *oskar* with the respective GFP-tagged proteins was calculated using an object-based nearest-neighbor analysis with xsColoc (Gaspar et al., 2017a) and choosing a colocalization window of 250 nm (consistent with the resolution limit of confocal microscopy). In this approach, image ‘segmentation’ was done to distinguish objects of interest from background in the two channels independently. This was followed by calculation of the distance between the nearest-neighbor objects using RNA as the reference channel and protein as the target channel. This was done within a confined relevant area of the image which in our case included the cytoplasm for the oocyte (follicle cells excluded) and cytoplasm and perinuclear regions for nurse cells (nurse cell and oocyte nuclei excluded). For statistical robustness, 100 iterations of random colocalization simulation were carried out in the confined area (as described above) using objects from the target channel. Colocalization frequency is expressed as the difference between observed and randomized frequencies for a defined colocalization window (250 nm). Note that due to particle crowdedness *in vivo*, probability of random colocalization is high, which leads to an underestimation of true colocalization (Gaspar et al., 2017a).

#### FRAP Analysis

FRAP movies were analyzed and intensity values for the pre-bleached and post-bleached ROI were obtained using Fiji. Analysis was done in FRAP Analyzer using double normalization and scaling the dynamic range from 0 to 1. Recovery curves were fitted with single exponential recovery using the formula:

FRAP(t) = I_0_ + I_1_.[1-e^-(t-t^bleach^)/ τ^ ] where, I_0_ = normalized intensity just after bleach and I_1_ = dynamic range of recovery. The fitted data has been plotted using Prism 8. The immobile fraction was calculated as (1- I_0_- I_1_)/(1- I_0_). The rate of recovery depends on the parameter τ from where the half-life of recovery (t_1/2_) is obtained as t_1/2_ = τ^.^ln2.

#### Calculation of partition coefficients

RFP-PTB partition coefficient (Figure 3C): For calculation of partition coefficient of RFP-PTB in Bruno and Hrp48 condensates, the Bruno or Hrp48 channels were segmented in Fiji and masks of the segmented particles were used to calculate mean intensities in the PTB channel. PC was calculated as the ratio of mean intensity inside condensates to that of the dilute phase.

*oskar* RNA partition coefficient in oocytes (Figure S5I): smFISH signal always appears as discrete puncta with the potential to detect single RNA molecules. When multiple RNA molecules are packaged together, as in case of *oskar* granules in the oocyte, the signal of the puncta appears brighter. Intensity of a single RNA puncta can be used to quantify the number of molecules in a granule. Segmentation of granules from the dilute phase/cytoplasm is done using intensity-based segmentation approaches. However, in the case of **Δ**N-EGFP, the diffuse, dim signal of *oskar* (in addition to some high intensity discrete puncta) indicates the presence of RNA that is not packaged into granules. We therefore assessed different intensity-based segmentation algorithms in Fiji and found that Intermodes (Prewitt and Mendelsohn, 1966) allows us to reliably distinguish granules from the diffuse *oskar* signal in the cytoplasm in the case of **Δ**N-EGFP. The granules were segmented using Intermodes algorithm in Fiji and PC calculated as the ratio of mean intensity inside granules to that of the cytoplasm. Single confocal sections were used for all the analysis.

#### Cortical analysis

For cortical analysis, CortAnalysis Fiji plugin was used (refer to Gaspar et al., 2014). The observed center of mass of *oskar* FISH signal was subtracted from the expected center of mass along the AP axis and the resultant value plotted.

#### Quantification of Oskar protein levels

For quantification of the Oskar protein signal in Figure 7 and related Figure S8, the region containing the signal was manually selected using a polygon tool and the integrated density quantified. The analysis was repeated for several oocytes of the same stage and the data plotted using Prism 8.

#### Statistical analysis

For all quantifications described above, statistical analyses were performed and data plotted using GraphPad Prism8. P-values were calculated using unpaired two-tailed t-test. In the figures, ^∗^ = p< 0.05, ^∗∗^ = p< 0.01, ^∗∗∗^ = p< 0.001, ^∗∗∗∗^ = p< 0.0001.

## Data Availability

• All fluorescence time-lapse movies reported in the study are available at the BioImage Archive (https://www.ebi.ac.uk/biostudies/studies/S-BIAD283). • Tomograms generated in this study are deposited in Electron Microscopy Data Bank (EMDB) under the following accession codes: EMDB:14212 https://www.ebi.ac.uk/emdb/EMD-14212, EMDB:14213 https://www.ebi.ac.uk/emdb/EMD-14213, EMDB:14214 https://www.ebi.ac.uk/emdb/EMD-14214, EMDB:14215 https://www.ebi.ac.uk/emdb/EMD-14215, EMDB:14216 https://www.ebi.ac.uk/emdb/EMD-14216, EMDB:14217 https://www.ebi.ac.uk/emdb/EMD-14217. • Any additional information required to reanalyze the data reported in this work paper is available from the Lead Contact upon request.
